# Vascular Damage in the Aorta of Wild-Type Mice Exposed to Ionizing Radiation: Sparing and Enhancing Effects of Dose Protraction

**DOI:** 10.3390/cancers13215344

**Published:** 2021-10-25

**Authors:** Nobuyuki Hamada, Ki-ichiro Kawano, Takaharu Nomura, Kyoji Furukawa, Farina Mohamad Yusoff, Tatsuya Maruhashi, Makoto Maeda, Ayumu Nakashima, Yukihito Higashi

**Affiliations:** 1 Radiation Safety Unit, Biology and Environmental Chemistry Division, Sustainable System Research Laboratory, Central Research Institute of Electric Power Industry (CRIEPI), Tokyo 201-8511, Japan; nomura@criepi.denken.or.jp; 2 Department of Cardiovascular Regeneration and Medicine, Research Institute for Radiation Biology and Medicine, Hiroshima University, Hiroshima 734-8551, Japan; kawano@hiroshima-u.ac.jp (K.-i.K.); drfarinamyusoff@hiroshima-u.ac.jp (F.M.Y.); maru0512@hiroshima-u.ac.jp (T.M.); 3 Biostatistics Center, Kurume University, Kurume 830-0011, Japan; furukawa_kyoji@med.kurume-u.ac.jp; 4 Natural Science Center for Basic Research and Development, Hiroshima 739-8526, Japan; mmaeda@hiroshima-u.ac.jp; 5 Department of Stem Cell Biology and Medicine, Graduate School of Biomedical and Health Sciences, Hiroshima University, Hiroshima 734-8551, Japan; ayumu@hiroshima-u.ac.jp; 6 Division of Regeneration and Medicine, Medical Center for Translational and Clinical Research, Hiroshima University Hospital, Hiroshima 734-8551, Japan

**Keywords:** ionizing radiation, aorta, vascular damage, inflammation, intima-media thickening, fibrosis, left ventricular function, C57BL6/J, ApoE^−/−^, aged mice

## Abstract

**Simple Summary:**

The circulatory system receives ionizing radiation at various dose rates. Here, we analyzed changes in the circulatory system of wild-type mice at six months after starting acute, intermittent or continuous irradiation with 5 Gy of photons. Irradiation had little effect on left ventricular function, heart weight, and kidney weight. In the aorta, acute exposure caused structural disorganizations and detachment of the aortic endothelium and intima-media thickening. These morphological changes were concomitant with increases in markers for profibrosis, fibrosis, proinflammation, and macrophages, along with decreases in markers for cell adhesion and vascular functionality in the aortic endothelium. Compared with acute exposure, the magnitude of such aortic changes was overall greater in 25 fractions, smaller in 100 fractions, and much smaller in chronic exposure. These findings suggest that dose protraction alters aortic vascular damage, in a way that is not a simple function of dose rate.

**Abstract:**

During medical (therapeutic or diagnostic) procedures or in other settings, the circulatory system receives ionizing radiation at various dose rates. Here, we analyzed prelesional changes in the circulatory system of wild-type mice at six months after starting acute, intermittent, or continuous irradiation with 5 Gy of photons. Independent of irradiation regimens, irradiation had little impact on left ventricular function, heart weight, and kidney weight. In the aorta, a single acute exposure delivered in 10 minutes led to structural disorganizations and detachment of the aortic endothelium, and intima-media thickening. These morphological changes were accompanied by increases in markers for profibrosis (TGF-β1), fibrosis (collagen fibers), proinflammation (TNF-α), and macrophages (F4/80 and CD68), with concurrent decreases in markers for cell adhesion (CD31 and VE-cadherin) and vascular functionality (eNOS) in the aortic endothelium. Compared with acute exposure, the magnitude of such aortic changes was overall greater when the same dose was delivered in 25 fractions spread over 6 weeks, smaller in 100 fractions over 5 months, and much smaller in chronic exposure over 5 months. These findings suggest that dose protraction alters vascular damage in the aorta, but in a way that is not a simple function of dose rate.

## 1. Introduction

In medical (therapeutic or diagnostic) and occupational settings, the circulatory system receives ionizing radiation exposure at various doses and dose rates. There has been a recent resurgence of interest in radiation effects on the circulatory system because a growing body of epidemiological evidence suggests that radiation exposure induces diseases of the circulatory system (DCS) at doses and dose rates much lower than previously considered [1,2,3]. Currently, the International Commission on Radiological Protection (ICRP) recommends a single nominal dose threshold for cardio- and cerebrovascular diseases independent of dose rate [4]. Dose rate dependence, manifestations, and mechanisms of such diseases, nonetheless, remain incompletely understood [5,6], and a better understanding is pivotal from viewpoints of radiation oncology and radiation protection.

Our previous study showed that vascular damage can occur in the aorta of wild-type C57BL6/J (B6J) mice at 1–6 months after a single acute exposure to 5 Gy of ^137^Cs γ-rays [7]. The present study aims to further investigate the impact of dose protraction on the circulatory system (the aorta in particular) at 6 months after starting irradiation with 5 Gy. To this end, we employed four additional irradiation regimens (a single acute exposure to X-rays, X-rays delivered in 25 fractions spread over 6 weeks, X-rays in 100 fractions spread over 5 months, and a chronic, low-dose-rate exposure to ^137^Cs γ-rays continuously over 5 months. The regimen with 25 fractions is relevant to radiotherapy patients who receive the dose of ≥5 Gy of X-rays (e.g., via thoracic irradiation and re-irradiation for lung cancer [8,9]) delivered conventionally in 20–30 fractions over 5–6 weeks (5 fractions/week). The regimen with 100 fractions is relevant to the patients treated with lung collapse for tuberculosis (TB) who on average received on the order of 100 chest X-ray fluoroscopic examinations over several years (cumulative dose up to ~18 Gy) [10]. The regimen with chronic exposure is relevant to some nuclear workers whose chronic cumulative dose exceeded 5 Gy [11]. For comparison, we also included a series of non-irradiated groups such as aged mice and apolipoprotein E-deficient (ApoE^−/−^) mice as positive controls each for senescent and DCS phenotypes, respectively. This was because irradiation often induces a premature (accelerated) senescence-like phenotype (e.g., in vascular endothelial cells (VECs) [12,13]), a senescent phenotype is involved in DCS etiology (e.g., in arterial disease [14]), and wild-type mice exhibit prelesional changes in the circulatory system (i.e., changes preceding DCS lesion formation), but not lesional changes (i.e., DCS phenotype).

To the best of our knowledge, this study is the first to report that dose protraction elicits both sparing and enhancing effects on vascular damage in the aorta. Prelesional changes observed in irradiated B6J mice were overall similar to those observed in non-irradiated but aged B6J mice or ApoE^−/−^ mice.

## 2. Materials and Methods

### 2.1. Mice and Shipping

Figure S1 depicts experimental timelines. For irradiation experiments at CRIEPI, B6J mice at 7 weeks of age were shipped by car (~48 km, ~1.2 h) from Charles River Laboratories Japan (Kanagawa, Japan) to CRIEPI (Tokyo, Japan), and were acclimated for a week prior to irradiation. At 5.2–5.5 months after starting irradiation, mice were shipped by car and air (~717 km, 6.6 ± 0.2 h) from CRIEPI to Hiroshima University (Hiroshima, Japan), followed by a two-week quarantine period. For irradiation experiments at Hiroshima University, B6J mice at age 7 weeks were shipped by car (~424 km, ~5.8 h) from Charles River Laboratories Japan (Shiga, Japan) to Hiroshima University, and were acclimated for a week prior to irradiation. For non-irradiated controls, B6J mice at age 5 weeks and 101 weeks were shipped by car (~778 km, ~10 h) from Charles River Laboratories Japan (Kanagawa) to Hiroshima University and were acclimated for three weeks before sampling. ApoE^−/−^ mice on the B6J genetic background purchased from Charles River Laboratories Japan were bred in-house, none of which experienced shipping during the course of experiments.

All mice used were male and maintained under a 12h light/dark cycle (light onset at 8 am) with ad libitum access to food and water. All mice were fed a normal-fat diet (NFD), except that two of four groups of ApoE^−/−^ mice aged 8 weeks were fed a high-fat diet (HFD) for 16 or 32 weeks. NFD used at CRIEPI was CE-2 (~3.4 kcal/g, ~4.6% of the calorie from crude fat) obtained from CLEA Japan. NFD and HFD used at Hiroshima University were MF (~3.5 kcal/g, ~13% of the calorie from crude fat) and HFD-60 (~5 kcal/g, ~60% of the calorie from crude fat), respectively, both of which were obtained from Oriental Yeast, Japan. Beddings used were paper chips (PaperClean, Japan SLC, Shizuoka, Japen) at CRIEPI and wood chips (Beta Chip, Northeastern Products Corp, Warrensburg, NY, USA) at Hiroshima University.

### 2.2. Irradiation and Sampling

This study consists of five irradiation regimens, all with 5 Gy. Just before irradiation, 8–10 unanesthetized B6J mice at age 8 weeks were placed in a 12-compartment pie cage (Natsume Seisakusho, Japan), except for mice receiving chronic γ-ray exposure in routine cages. At CRIEPI, mice were exposed to X-rays (260 kVp and 4.5 mA with a 0.5 mm Al and 0.3 mm Cu filter, at a source-surface distance of 52.7 cm), as a single acute dose, or intermittently in 25 daily fractions (0.2 Gy/fraction spread over 42 days) or in 100 fractions (0.05 Gy/fraction spread over 153 days) from a Faxitron MultiRad350 irradiator at a dose rate of 0.5 Gy/min, or continuously exposed to ^137^Cs γ-rays over 153 days at a dose rate of <1.4 mGy/h (at a source-surface distance of 371 cm). We designate the last regimen “chronic” irradiation, as mice were continuously exposed except for a time needed for husbandry (67.6 h in 153 days, 3.1 h/week). At Hiroshima University, mice were exposed to a single acute dose of ^137^Cs γ-rays from Gammacell 40 Exactor at a dose rate of 0.5 Gy/min, as described [7]. A pie cage was rotated on a turntable during X-ray exposure, but not during acute γ-ray exposure. Sham (0 Gy)-irradiated controls were handled in parallel with the test (5 Gy irradiated) mice.

At age 8 weeks for non-irradiated “young” B6J or ApoE^−/−^ mice, age 24 weeks for ApoE^−/−^ mice fed HFD for 16 weeks, age 34 weeks for irradiated or sham-irradiated B6J mice (i.e., at 6 months after starting irradiation), age 40 weeks for ApoE^−/−^ mice fed HFD for 32 weeks, and age 104 weeks (2 years) for non-irradiated “aged” B6J or ApoE^−/−^ mice, mice were weighed, anesthetized with isoflurane, subjected to echocardiography, and perfused transcardially with phosphate-buffered saline (PBS^−^), followed by tissue sampling. Of the collected descending thoracic aorta, the cranial half was evaluated for morphological changes with field-emission scanning electron microscopy (FE-SEM), the caudal half being assessed for cellular and molecular changes with immunofluorescence and histochemical (Masson’s trichrome and Oil Red O) staining. No B6J mice died or appeared moribund throughout the observation period. During the course of experiments, irradiated mice exhibited a slight but statistically significant decrease in body weight compared with sham-irradiated mice, and shipping from CRIEPI to Hiroshima caused a significant decrease in body weight in all groups (Figure S2). At tissue sampling, heart and kidneys were also weighed (Figure S3A–E). For each mouse, right and left kidneys were weighed together, then the mean was used as kidney weight.

### 2.3. Echocardiography

Anesthetized mice underwent motion/movement-mode (M-mode) echocardiography with a Toshiba Nemio MX SSA-590A ultrasound scanner and a Toshiba PLM-1202S ultrasound probe. Interventricular septal thickness at end diastole (IVSTd), left ventricular dimension at end diastole (LVDd), left ventricular posterior wall thickness at end diastole (PWTd), and left ventricular dimension at end systole (LVDs) were measured as presented in Figure S4A. Left ventricular ejection fraction (LVEF), left ventricular fractional shortening (LVFS), and left ventricular mass (LVM) were calculated as described [15].

### 2.4. FE-SEM

**Figure 1:**
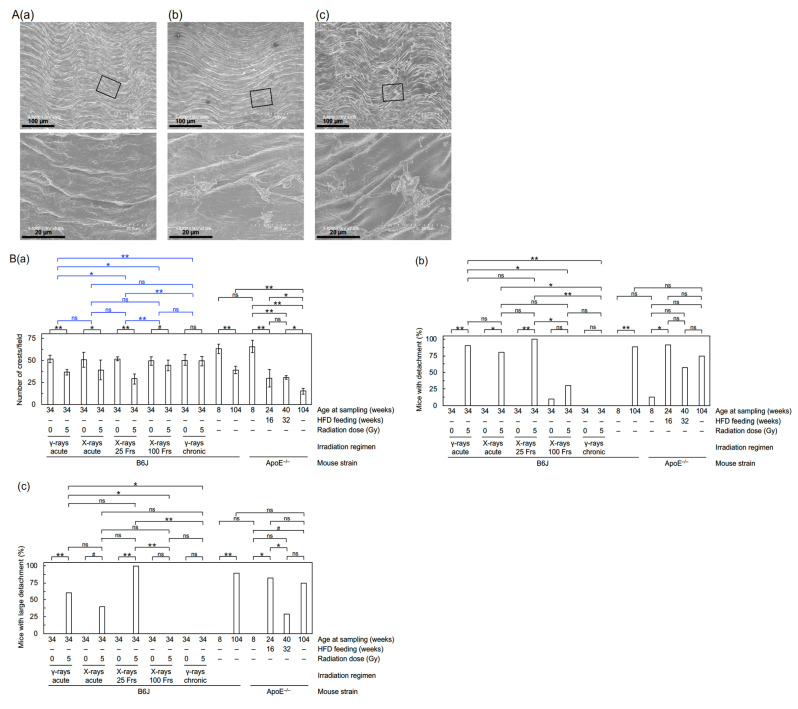
Morphological changes in the aortic endothelium. (**A**) Representative FE-SEM images of (**a**) normal endothelium (young mouse), and (**b**,**c**) detachment and large detachment, respectively (6 months after starting irradiation with 5 Gy of X-rays in 25 fractions), all in B6J mice. Boxed areas in the upper panels are shown at higher magnification in the lower panels. Scale bars as indicated. (**B**) Quantitative analysis for (**a**) the number of crests/field (7–11 mice/group analyzed except for 4 in aged ApoE^−/−^ mice, Welch’s *t*-test), and (**b**,**c**) percentage of mice with detachment and large detachment, respectively (7–11 mice/group analyzed except for 20 in B6J mice at 6 months after irradiation with 0 Gy or 5 Gy of acute γ-rays and 4 in aged ApoE^−/−^ mice, Fisher’s exact test). For an outline of statistical comparisons, see a footnote in Supplementary Materials. Frs, fractions. The data in Figure 1B(**a–c**) for the two B6J groups receiving 0 Gy or 5 Gy of acute γ-rays were taken from the 2020 Cancers paper [7]. **, *p* < 0.001. *, 0.001 ≤ *p* < 0.05. #, 0.05 ≤ *p* < 0.1 (marginally significant). ns, *p* ≥ 0.1 (nonsignificant).

The cranial half of the descending thoracic aorta was opened longitudinally, fixed and carbon coated, followed by the FE-SEM analysis of the entire area, as described [7]. The surface of the normal aortic endothelium exhibited less frequent horizontal waves and more frequent vertical waves (Figure 1A(a)), and the number of crests in such vertical waves was counted in each of the seven fields/mouse (each field corresponds to the entire area of the image taken at 300 × magnification, 1 crest/field corresponding to ~7.5 crests/mm^2^). The detached area in the aortic endothelium with sizes in the longer axis of a few tens of microns was designated “detachment” (Figure 1A(b)) and that of the order of 100 µm was designated “large detachment” (Figure 1A(c)). Each mouse was considered positive if one or more such areas existed in the aorta, and such positivity in the group was evaluated independent of the number of such areas in each mouse (this was also the case for rolling leukocytes).

### 2.5. Immunofluorescence and Histochemistry

The caudal half of the descending thoracic aorta was embedded, snap-frozen, and transversally cryosectioned at a 5 µm thickness for staining, and the section was mounted onto a glass slide, as described [7].

Dual immunofluorescence was performed for cluster of differentiation 31 (CD31)/platelet endothelial cell adhesion molecule 1 (PECAM-1) stained green and one of the other markers stained red, with cell nuclei counterstained with 4’,6-diamidino-2-phenylindole (DAPI), as described [7]. Staining with primary antibodies against CD31, endothelial nitric oxide synthase (eNOS), vascular endothelial cadherin (VE-cadherin), tumor necrosis factor α (TNF-α), and F4/80 and secondary antibodies (anti-rabbit and anti-rat) was quantified as described [7]. For transforming growth factor β1 (TGF-β1), CD68, and CD3, primary antibodies used were all rabbit polyclonal and purchased from Sigma (TGF-β1) and Abcam (CD68 and CD3). For each mouse, staining was quantified as follows: intensity of red signals in randomly selected five areas (50 µm × 50 µm) in the tunica media in one image taken at 20 × magnification for TGF-β1, the number of dots in the entire aortic area (including the tunica adventitia) in four images taken at 20 × magnification for CD68 and CD3. In seven tiled images for each mouse, a maximum distance between the inner (luminal) side of the tunica intima and the outer side of the tunica media was measured as the intima-media thickness (IMT).

Following the instructions from the manufacturer (Muto Pure Chemicals, Tokyo, Japan), Masson’s trichrome staining was conducted where aniline blue stains collagen fibers in the tunica media blue. Arterial wall area, intensity of aniline blue staining, and IMT were measured in a single tiled image for each mouse.

Oil Red O stains neutral lipids red, thereby visualizing atherosclerotic plaques in the aorta. Oil Red O staining was performed using a kit (Cat. No. ORK-1-IFU, ScyTek Laboratories, Logan, UT, USA) according to the manufacturer’s instructions, with cell nuclei counterstained with Mayer’s hematoxylin (Lillie’s modification).

### 2.6. Statistical Analysis

Statistical analyses were performed using R statistical software (version 3.6.1, R Foundation, https://www.r-project.org/, last accessed 25 October 2021), where a *p*-value (after Bonferroni corrections for pairwise comparisons using the *t*-test) of < 0.05 was considered significant (*p* < 0.001 presented as **, 0.001 ≤ *p* < 0.05 as *), 0.05 ≤ *p* < 0.1 as marginally significant (presented as ^#^) and *p* ≥ 0.1 as nonsignificant (presented as ns). Black asterisks or pound signs were used for intra-regimen comparisons (e.g., irradiated vs. sham-irradiated groups in each irradiation regimen), whereas blue asterisks or pound signs were used for inter-regimen comparisons of such intra-regimen differences (i.e., the degree of differences in irradiated and sham-irradiated groups between the two regimens). *p* values determined by the one-way analysis of variance (ANOVA) using the F-test of homogeneity among the group means are presented as ANOVA *p*. *p* values determined by the two-sample (Welch’s) *t*-test for the null hypothesis of equal means, chi-square test, Fisher’s exact test, Wald test (logistic regression), and Kolmogorov–Smirnov goodness-of-fit test are presented as *p*. The statistical test used is described in figure legends or table footnotes. Each data point was obtained from 7–11 mice (but 4 in aged ApoE^−/−^ mice) and represents means and standard deviations, unless otherwise specified.

## 3. Results

This study is composed of ≤16 groups (12 groups of B6J mice and 4 groups of ApoE^−/−^ mice), 33 prelesional endpoints (5 for body or organ weight, 8 for echocardiography, 4 for FE-SEM of the aorta, 12 for immunofluorescence of the aorta and 4 for Masson’s trichrome staining of the aorta), and statistical comparisons for ≤24 pairs in each endpoint. An outline of statistical comparisons for each endpoint given in each figure legend is not repeated here, and the following subsections first explain the results of 10 irradiated or sham-irradiated groups of B6J mice for the impact of radiation, and then on the results of 2 non-irradiated groups of B6J mice and 4 non-irradiated groups of ApoE^−/−^ mice for the impact of aging and ApoE deficiency.

### 3.1. Radiation

**Figure 2:**
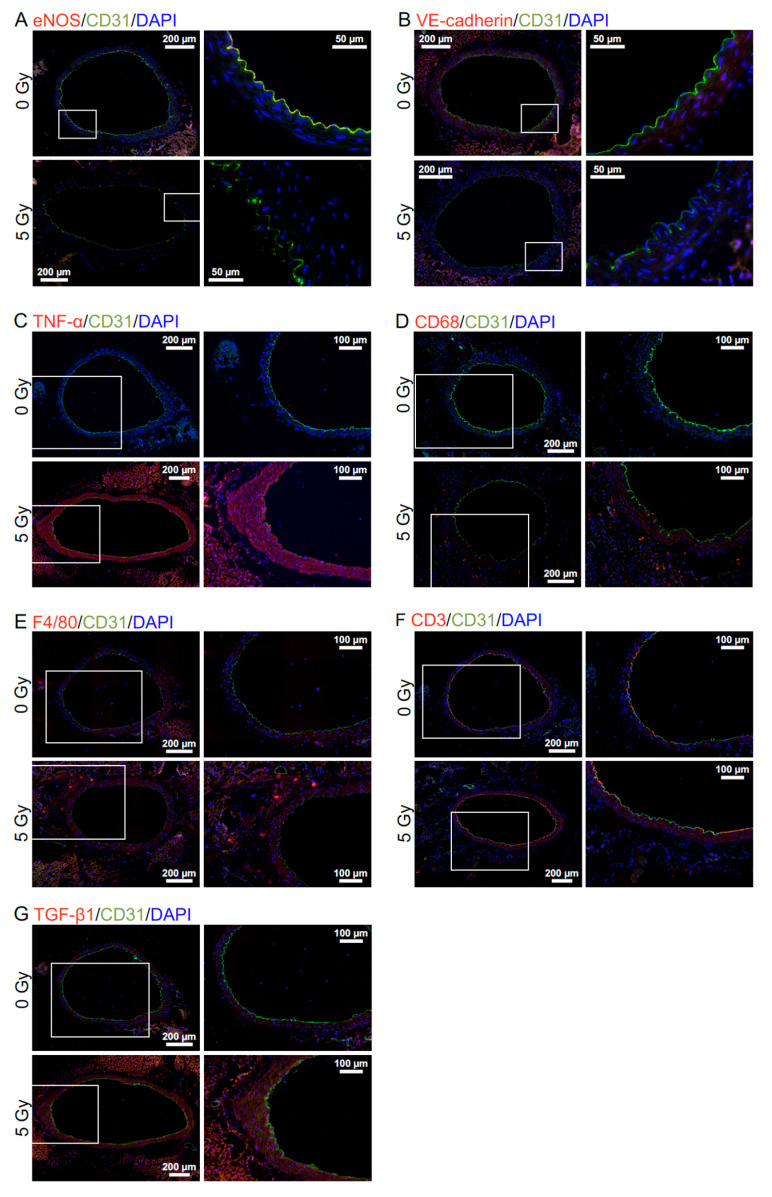
Molecular changes in the aorta. Representative merged images in B6J mice (all at 6 months after starting irradiation) for double immunofluorescence of CD31 with (**A**) eNOS, (**B**) VE-cadherin, (**C**) TNF-α, (**D**) CD68, (**E**) F4/80, (**F**) CD3 or, (**G**) TGF-β1, with cell nuclei counterstained with DAPI. (**A**,**C**,**E**–**G**) X-rays in 25 fractions. (**B**,**D**) X-rays in 100 fractions. Upper panels, 0 Gy. Lower panels, 5 Gy. Boxed areas in the left panels (tiled images) are shown at higher magnification in the right panels. Scale bars as indicated.

**Figure 3:**
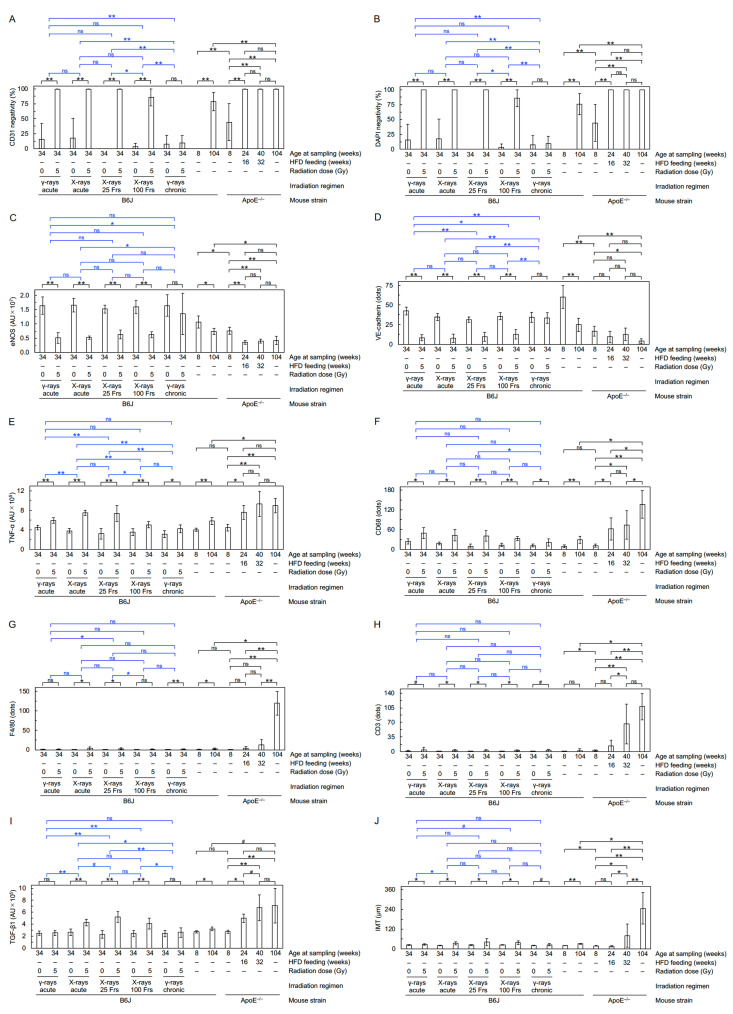
Molecular changes in the aorta. Quantitative analysis of immunofluorescence for (**A**) CD31 negativity, (**B**) DAPI negativity, (**C**) eNOS, (**D**) VE-cadherin, (**E**) TNF-α, (**F**) CD68, (**G**) F4/80, (**H**) CD3, (**I**) TGF-β1, and (**J**) IMT (7–11 mice/group analyzed except for 4 in aged ApoE^−/−^ mice, Welch’s *t*-test, or Wald test). For clarity, graphs replotted for F4/80, CD3, and IMT in B6J mice only are shown in Figure S6A–C). For an outline of statistical comparisons, see a footnote in Supplementary Materials. AU, arbitrary unit. Frs, fractions. The data in Figure 3A–E,G) for the two B6J groups receiving 0 Gy or 5 Gy of acute γ-rays were taken from the 2020 Cancers paper [7]. Representative images in B6J mice are shown in Figure 2. **, *p* < 0.001. *, 0.001 ≤ *p* < 0.05. #, 0.05 ≤ *p* < 0.1 (marginally significant). ns, *p* ≥ 0.1 (nonsignificant).

**Figure 4:**
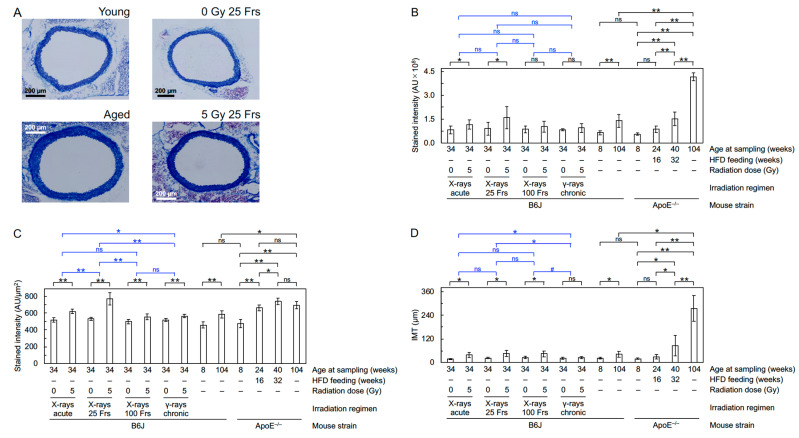
Fibrotic changes in the aorta. (**A**). Representative images for Masson’s trichrome staining in B6J mice (young, aged, at 6 months after starting irradiation with 0 Gy or 5 Gy of X-rays in 25 fractions). Quantitative analysis for (**B**) total intensity of aniline blue stain in the entire aortic wall, (**C**) intensity of aniline blue stain per unit aortic wall area, and (**D**) IMT (8–10 mice/group analyzed except for 4 in aged ApoE^−/−^ mice, Welch’s *t*-test). For clarity, a graph replotted for IMT in B6J mice only is shown in Figure S9C. For an outline of statistical comparisons, see a footnote in Supplementary Materials. AU, arbitrary unit. Frs, fractions. **, *p* < 0.001. *, 0.001 ≤ *p* < 0.05. #, 0.05 ≤ *p* < 0.1 (marginally significant). ns, *p* ≥ 0.1 (nonsignificant).

At 6 months after starting irradiation, irradiated B6J mice showed differences in 24 of 33 endpoints (16 increased, 8 decreased) at least in one irradiation regimen, compared with sham-irradiated B6J mice (Table S1). Of these, for body or organ weight and echocardiography (Figures S3A–E and S4B), there were slight differences in 6 of 13 endpoints (1 increase, 5 decreased) in ≤3 of 5 irradiation regimens (a decrease in body weight and an increase in kidney weight/body weight in the “acute γ-rays” regimen, decreases in body weight, LVDd and LVM in the “acute X-rays” regimen, decreases in body weight, kidney weight and PWTd in the “chronic γ-rays” regimen). In contrast, for the aorta (Figure 1, Figure 2, Figure 3, Figure 4 and Figures S5–S10), there were differences in 18 of 20 endpoints (all but mice with rolling leukocytes and aortic wall area) in ≤5 irradiation regimens, and the following two paragraphs in this subsection thence focus on the aorta.

First, FE-SEM analysis was conducted to evaluate the impact on the morphology of the aortic endothelium. The aortic surface in young mice showed a morphology of undulations with regular repeating (Figure 1A(a)), which was disturbed in irradiated mice. Four irradiation regimens (except for chronic γ-rays) reduced waviness (Figure 1B(a)) that was considered attributable to various accompanying morphological changes, such as flattening, derangement, and cobblestone formation (Figure S5A(a–c)). Three irradiation regimens (acute γ-rays, acute X-rays, and X-rays in 25 fractions) led to an increase in detachment and a large detachment of the aortic surface (Figure 1B(b,c)). These findings indicate radiation-induced morphological vascular damage. Second, immunofluorescent staining was performed to assess molecular changes (Figure 2A–G). In VECs, four irradiation regimens (except for chronic γ-rays) resulted in decreases in eNOS (a vascular functionality marker) and VE-cadherin (an adherens junction marker) and increases in CD31 negativity (indicating loss of VECs or CD31), DAPI negativity (indicating loss of VECs) and subcellular fragments (indicative of apoptosis) (Figure 3A–D, Figure S8A,B(a)). This, along with the observation of detachment and large detachment (Figure 1B(b,c)), suggests that irradiation induces vascular damage manifested as partial loss of aortic endothelium whose mechanisms involve VEC apoptosis. In vascular smooth muscle cells (VSMCs), 3–5 irradiation regimens increased TNF-α (a proinflammation marker), CD68 and F4/80 (macrophage markers), CD3 (a T-cell marker), and subcellular fragments (indicative of apoptosis) (Figure 3E–H, Figures S6A,B and S8A,B(b)). This, along with the observation of rolling leukocytes (Figure S5A(d),B), suggests that radiation-induced vascular damage induces inflammation. Reportedly, TNF-α (and other factors such as ADAM10) mediates degradation and internalization of VE-cadherin, which in turn increases vascular permeability to macromolecules [16,17,18]. In the aortic wall, 3–5 irradiation regimens increased TGF-β1 (a profibrosis marker), aniline blue stain (a collagen fiber marker), and IMT (with no difference in IMT determined by two approaches in any group) (Figure 3I,J, Figure 4A–D, Figures S6C and S9A–D), suggesting that irradiation promotes fibrosis. Altogether, the present data suggest that irradiation causes vascular damage and dysfunction, inflammation, and fibrosis, all of which are thought to be involved in the early stages of atherosclerosis.

Among 18 endpoints that showed differences between irradiated and sham-irradiated B6J mice at least in one irradiation regimen, 16 endpoints showed differences at least in one pair among irradiation regimens (Table S2). Based on these 16 endpoints, we took two integrative approaches to compare the biological effectiveness of different irradiation regimens. In the first approach (Table S2), four different levels of difference (presented as ns, #, * and ** in the graphs for each endpoint) were converted to discrete scores (0, ±0.5, ±1, and ±1.5). In the second approach (Table S3), Kolmogorov–Smirnov goodness-of-fit test was employed. Both approaches suggested effectiveness in a descending order of X-rays in 25 fractions > acute X-rays > acute γ-rays > X-rays in 100 fractions >> chronic γ-rays.

### 3.2. Aging and ApoE Deficiency

Compared with young B6J mice, aged B6J mice showed differences in 28 endpoints (25 increased, 3 decreased) (Table S4). Between irradiated and aged B6J mice, 18 endpoints (14 increased, 4 decreased) of 28 (aged vs young B6J mice, 25 increased, 3 decreased) or of 24 (irradiated vs sham-irradiated B6J mice, 16 increased, 8 decreased) endpoints were common in the same direction (Table S5). This indicates that in B6J mice, many radiogenic prelesional changes are relatively akin to age-related changes albeit in slightly different magnitude.

There were differences in 22 endpoints (18 increased, 4 decreased) between young ApoE^−/−^ mice and aged ApoE^−/−^ mice (Table S6), and 20 endpoints (17 increased, 3 decreased) of 28 (aged vs young B6J mice, 25 increased, 3 decreased) or of 22 endpoints (aged vs young ApoE^−/−^ mice, 18 increased, 4 decreased) were common in the same direction between aged B6J mice and aged ApoE^−/−^ mice (Table S7). There were differences in 17 endpoints (11 increased, 6 decreased) between young B6J mice and young ApoE^−/−^ mice (Table S8), and in 17 endpoints (13 increased, 4 decreased) between aged B6J mice and aged ApoE^−/−^ mice (Table S9). This indicates that B6J mice and ApoE^−/−^ mice fed with NFD have distinct phenotypes already at age 8 weeks and show similar age-related prelesional changes but in varying magnitude.

All 14 groups mentioned above were fed NFD, but the two groups of ApoE^−/−^ mice were fed HFD from age 8 weeks onwards. There were differences in 17 endpoints (12 increased, 5 decreased) between young ApoE^−/−^ mice and ApoE^−/−^ mice fed HFD for 16 weeks (Table S10), in 13 endpoints (10 increased, 3 decreased) between ApoE^−/−^ mice fed HFD for 16 weeks and ApoE^−/−^ mice fed HFD for 32 weeks (Table S11), and in 14 endpoints (12 increased, 2 decreased) between ApoE^−/−^ mice fed HFD for 32 weeks and aged ApoE^−/−^ mice (Table S12). This highlights differences posed by HFD feeding and aging in ApoE^−/−^ mice.

Last, as a lesional change, ApoE^−/−^ mice fed HFD for 32 weeks and aged ApoE^−/−^ mice, but not other 14 groups, developed atherosclerotic plaques in the aorta that were characterized by its typical morphological features and were positive with markers for macrophages (CD68 and F4/80) and neutral lipids (Oil Red O) (Figures S7 and S10B).

## 4. Discussion

We have here reported the effects on the circulatory system in 12 groups of B6J mice and in 4 groups of ApoE^−/−^ mice, for various endpoints, i.e., weight of the heart and kidneys (Figure S3), echocardiographic changes (Figure S4), morphological and molecular changes in the aorta (Figure 1, Figure 2, Figure 3, Figure 4 and Figures S5–S10). The following two subsections deal first with radiation, and then aging and ApoE deficiency, while recognizing the possibility that other factors may also have affected the results, such as inter-group differences in potential stress posed by shipping (in a range of 48–778 km in 1.2–10 h by car or air), as well as inter-institutional differences in NFD as regular chow (4.6% vs 13% of the calorie from crude fat) and in beddings (paper chips vs wood chips).

### 4.1. Radiation

It has been reported that at 20 weeks after local heart irradiation with a single acute dose of 2 Gy of 200 kV X-rays (0.8 Gy/min), irradiated male B6J mice exhibited significant echocardiographic changes (decreases in LVEF and LVFS, increases in LVDd, IVSTd, LVM/body weight), compared with sham-irradiated counterparts, with no difference in heart weight/body weight [19]. In this respect, we conducted weight measurements and echocardiography in irradiated male B6J mice at 6 months (~26 weeks) after starting acute, intermittent, or chronic exposures of the whole body to 5 Gy of 260 kVp X-rays or ^137^Cs γ-rays. We found that there were slight differences in the “acute X-rays” regimen for two endpoints (LVDd and LVM) and in the “chronic γ-rays” regimen for four endpoints (kidney weight, kidney weight/body weight, IVSTd and PWTd), and there was no heterogeneity for any endpoint in the degree of a difference between irradiated and sham-irradiated groups among irradiation regimens (Figures S3 and S4). Given that the present data exhibited such little radiogenic changes, the discussion below focuses on radiogenic changes in the aorta, particularly regarding impacts of radiation quality and dose protraction because our two integrative approaches both suggested biological effectiveness in descending order of X-rays in 25 fractions > acute X-rays > acute γ-rays > X-rays in 100 fractions >> chronic γ-rays (Tables S2 and S3).

#### 4.1.1. Higher Biological Effectiveness of Acute X-rays vis-à-vis Acute γ-rays

Tables S2 and S3 suggest that acute exposure to 260 kVp X-rays is more effective than that to ^137^Cs γ-rays. Here, it is noteworthy that given the same entrance skin dose (i.e., 5 Gy in this study), depth dose is lower for 260 kVp X-rays than ^137^Cs γ-rays, so biological effectiveness in vivo of 260 kVp X-rays vis-à-vis ^137^Cs γ-rays per unit dose can be higher when based on the dose at the organ of interest (i.e., aorta in this study) than based on entrance skin dose. This finding is consistent with a radiobiological tenet that the lower the energy of photons (X-rays and γ-rays), the higher the biological effectiveness [20,21]: specifically, kilovoltage X-rays (a hundred or a few keV) are known to be ~1.5-fold more effective than ^137^Cs γ-rays (662 keV) in vitro [22] and in vivo [23]. The interesting observation of increases in TGF-β1 and F4/80 following acute X-rays but with no changes following acute γ-rays (Figure 3G,I) necessitates further study.

#### 4.1.2. Enhancing Dose Protraction Effect following X-rays in 25 Fractions vis-à-vis Acute X-rays and Acute γ-rays

Tables S2 and S3 suggest that the “X-rays in 25 fractions” regimen is slightly more effective than the “acute X-rays” regimen and is much more effective than the “acute γ-rays” regimen. To the best of our knowledge, this is the first in vivo study to show such potential enhancing (inverse) dose protraction effects for vascular damage. In support, there is the in vivo observation in kidneys of B6J mice that exposures to 5 Gy of ^137^Cs γ-rays in 25 fractions tend to be more effective than a single acute dose at inducing senescence-associated β-galactosidase, mitochondrial DNA common deletion, and p21 expression [24]. An in vitro study with human umbilical vein endothelial cells also reported that exposures to 0.5 Gy of X-rays in two fractions led to increases in reactive oxygen species production, p65 phosphorylation (indicative of nuclear factor κB activation), intercellular adhesion molecule 1 induction, and adhesion to polymorphonuclear leukocytes, compared with a single acute dose [25]. These biological findings are further supported epidemiologically. For instance, in the Canadian TB fluoroscopy cohort of patients who received highly fractionated exposures to X-rays, the excess relative risk per unit dose (ERR/Gy) for ischemic heart disease (IHD) mortality increased with decreasing dose per year when a lag of 10 years was used, e.g., ERR/Gy (95% confidence intervals) of 0.01 (–0.043, 0.078) at 0.3–7.3 Gy/year, 0.145 (0.007, 0.32) at 0.15–0.29 Gy/year, and 0.592 (0.004, 1.4) at 0.0004–0.14 Gy/year [26]. A meta-analysis for IHD and cerebrovascular disease has also indicated larger risks per unit dose for fractionated and lower dose rate exposures [1]. Moreover, estimates of lifetime excess DCS mortality risk per unit dose predicted from the acutely exposed Japanese atomic bomb survivor Life Span Study data were nearly doubled by decreasing the dose from 1 Gy to 0.01 Gy [27], along with a somewhat similar in vivo observation for cardiovascular disease mortality following ^60^Co γ-ray exposure [28], indicative of inverse dose protraction effect.

#### 4.1.3. Sparing Dose Protraction Effect following X-rays in 100 Fractions and Chronic γ-rays vis-à-vis Acute X-rays and Acute γ-rays

Tables S2 and S3 suggest that the “X-rays in 100 fractions” regimen is less effective than “acute X-rays” and “acute γ-rays” regimens and is much less effective than the “X-rays in 25 fractions” regimen. This finding of sparing effects is consistent with another radiobiological tenet that the biological effectiveness of low-linear energy transfer (LET) radiation like photons decreases with decreasing dose rates [29]. Besides, Tables S2 and S3 suggest that the “chronic γ-rays” regimen is less effective than the “X-rays in 100 fractions” regimen, despite the same dose was delivered over the same period of time (i.e., 153 days) in these two regimens, whilst the possibility that radiation quality effect affects the results cannot be ruled out. Further experiments are required to address whether there is any “boundary” dose protraction regimen above which enhancing protraction effects occur, below which sparing effects occur (the present data suggests that such a boundary may exist somewhere between 25 and 100 fractions), and any “boundary” dose protraction regimen that produces no difference in effects with chronic exposure (the present data suggests such a boundary may exist in >100 fractions).

#### 4.1.4. Limitations

In addition to inter-group and inter-institutional differences as aforementioned, our experimental design of irradiation experiments has several limitations that may be improved in the future. First, the radiation source used for various irradiation regimens was inconsistent, e.g., without conducting fractionated exposures to ^137^Cs γ-rays owing in part to inter-institutional differences in infrastructures (i.e., a high dose rate irradiator available only at Hiroshima University vs a low dose rate irradiator available only at CRIEPI), although chronic X-ray exposure we did not conduct either is technically unfeasible. Second, the duration of irradiation for the “X-rays in 25 fractions” regimen (spread over 42 days) was inconsistent with the “X-rays in 100 fractions” and “chronic γ-rays” regimens (spread over 153 days). Third, age at acute X-ray or γ-ray exposure did not include 14 weeks (when delivery of X-rays in 25 fractions completes) and 30 weeks (when delivery of X-rays in 100 fractions and chronic γ-rays completes). Fourth, the dose response was not examined at multiple dose points (e.g., to include a low dose of 0.05 Gy, a moderate dose of 0.5 Gy). Last, temporal changes were not compared at multiple post-irradiation time points (e.g., to include a time point of 12 months after starting irradiation, whereas we previously looked at earlier time points of 1 and 3 months after a single acute dose of ^137^Cs γ-rays [7]).

A series of aortic changes observed following total body irradiation should result not only from direct effects to the aorta but also from effects from various organs/tissues (including the heart, kidneys, and other potential target organs/tissues for radiation effects on the circulatory system). Nevertheless, we consistently employed total body irradiation for all irradiation regimens because local irradiation is unfeasible for chronic, continuous exposures, and is impractical for highly fractionated exposures. Reportedly, highly fractionated partial heart irradiation is not unfeasible, but mice need to receive fluoroscopic X-rays repeatedly for positioning purposes ([30]).

### 4.2. Aging and ApoE Deficiency

Overall, various prelesional changes observed in irradiated B6J mice resembled those in aged B6J mice and ApoE^−/−^ mice (but in varying magnitude), although ApoE^−/−^ mice, but not B6J mice, showed lesional changes. This encourages further studies to investigate the modifying effects of age at exposure, attained age, time since exposure, sex, lifestyle (e.g., diet), environmental factors, genetics, epigenetics, strains/races, comorbidities, and other factors governing inter-individual or inter-population differences in radiation response [31]. In this regard, irradiation experiments in ApoE^−/−^ mice which we did not conduct in this study may provide insights: at a high dose rate (0.8 Gy/min, X-rays), premature death (half of the mice died by 20 weeks post-irradiation) was observed with altered cardiomyocyte structure and function at a moderate dose (0.2 Gy), but not at a high dose (2 Gy) [19]. Likewise, enhanced aortic atherosclerosis was observed at a moderate dose (0.2 Gy) after a high dose rate (0.89 Gy/min, X-rays), but not after a low dose rate (0.02 Gy/day, ^137^Cs γ-rays), and such enhanced aortic atherosclerosis was absent at a high dose (6 Gy) with no difference between low and high dose rates [32]. Conversely, hermetic responses (phenomena where low-level insult makes predisposed individuals healthier) have also been reported [33,34], e.g., reduced plaque size after moderate dose, low dose rate (0.157 Gy at 28 µGy/h) but not after low dose, low dose rate (0.067 Gy at 12 µGy/h) [34]. As such, it is evident that there is no consensus about the role of ApoE in radiation responses, necessitating further studies. Pertinently, the recent study reported atherogenesis in low-density lipoprotein receptor-deficient (Ldlr^−/−^) mice receiving highly fractionated exposures at moderate or high dose (0.5 Gy in 75 fractions at 0.067 Gy/1 min/fraction, or 1 Gy in 75 fractions at 0.134 Gy/2 min/fraction, both as 5 fractions/week over 15 weeks), but did not include the data for acute or continuous exposure [35], so the impact of LDLR on dose protraction remains elusive.

## 5. Conclusions

The present results suggest that irradiation of B6J mice causes vascular damage and dysfunction, inflammation and fibrosis in the aorta, all of which have been implicated in the early stages of atherosclerosis. Various prelesional changes observed in irradiated B6J mice were qualitatively similar to those in aged B6J mice or ApoE^−/−^ mice (but often quantitatively different), although ApoE^−/−^ mice, but not B6J mice, showed lesional changes. Our integrative approaches based on multiple aortic endpoints suggest that dose protraction alters vascular damage in the aorta, but in a way that is not a simple function of dose rate, i.e., biological effectiveness in descending order of X-rays in 25 fractions > acute X-rays > acute γ-rays > X-rays in 100 fractions >> chronic γ-rays. Identification of the underpinning mechanisms warrants further studies, which will be indispensable for consideration of implications for radiation oncology and radiation protection, and for developing biology-based dose-response models and adverse outcome pathways for DCS [36,37], including consideration of roles of immune cells [38]. With the endpoints identified in the present study for evaluation of prelesional changes, experiments are underway to investigate dose protraction effects in B6J mice at a later timepoint.

## Data Availability

The data presented in the current study are available from the first and corresponding author (N.H.) upon reasonable request.

## Supplementary Materials

The following are available online at https://www.mdpi.com/article/10.3390/cancers13215344/s1, Footnotes to Figure 1, Figure 3 and Figure 4, Figure S1. Experimental timelines, Figure S2. Temporal changes in body weight of irradiated or sham-irradiated B6J mice, Figure S3. Changes in body weight, heart weight and kidney weight at sampling, Figure S4. Changes in left ventricular function, Figure S5. Morphological changes in the aortic endothelium, Figure S6. Molecular changes in the aorta of B6J mice, Figure S7. Molecular changes in the aorta of ApoE^−/−^ mice, Figure S8. Nuclear changes in the aorta, Figure S9. Fibrotic changes in the aorta, Figure S10. Atherosclerotic plaques in the aorta, Figure S10. Atherosclerotic plaques in the aorta, Table S1. List of 24 endpoints changed in irradiated B6J mice at least in one irradiation regimen (vs. sham-irradiated B6J mice), Table S2. Comparison of multiple endpoints in relation to changes in the aorta by scoring of categorized levels of statistical differences among irradiation regimens, Table S3. Comparison of radiation effects in the aorta averaged over 16 endpoints among irradiation regimens, Table S4. List of 28 endpoints changed in aged B6J mice (vs. young B6J mice), Table S5. List of endpoints changed in irradiated B6J mice and aged B6J mice, Table S6. List of 22 endpoints changed in aged ApoE^−/−^ mice (vs. young ApoE^−/−^ mice), Table S7. List of endpoints changed in aged B6J mice and aged ApoE^−/−^ mice, Table S8. List of 17 endpoints changed in young ApoE^−/−^ mice (vs. young B6J mice), Table S9. List of 17 endpoints changed in aged ApoE^−/−^ mice (vs. aged B6J mice), Table S10. List of 17 endpoints changed in ApoE^−/−^ mice fed HFD for 16 weeks (vs. young ApoE^−/−^ mice), Table S11. List of 13 endpoints changed in ApoE^−/−^ mice fed HFD for 32 weeks (vs. ApoE^−/−^ mice fed HFD for 16 weeks), Table S12. List of 14 endpoints changed in aged ApoE^−/−^ mice (vs. ApoE^−/−^ mice fed HFD for 32 weeks).
