# Supplementary material for: Vascular Damage in the Aorta of Wild-Type Mice Exposed to Ionizing Radiation: Sparing and Enhancing Effects of Dose Protraction
Source: Cancers (Basel). 2021 Oct 25;13(21):5344. doi: 10.3390/cancers13215344 (PMC8582417; doi:10.3390/cancers13215344)
Supplement: Supplementary file 1 [file cancers-13-05344-s001.zip › cancers-1417312_Supplementary_Publish_Chk.pdf]

Supplementary Material

Vascular Damage in the Aorta of Wild-Type Mice Exposed to Ionizing Radiation: Sparing and Enhancing Effects of Dose Protraction

Nobuyuki Hamada, Ki-ichiro Kawano, Takaharu Nomura, Kyoji Furukawa, Farina Mohamad Yusoff, Tatsuya Maruhashi, Makoto Maeda, Ayumu Nakashima and Yukihito Higashi

Footnotes to Figures 1, 3 and 4

A footnote to Figure 1B. (a,b,c) Among five sham-irradiated groups, there was no heterogeneity for one endpoint (crests, ANOVA $p = 0.8$), though such differences were untestable for two endpoints (detachment, large detachment). Among five irradiation regimens, one endpoint (crests) was lower in four regimens (except for chronic γ-rays), and two endpoints (detachment, large detachment) were higher in three regimens (acute γ-rays, acute X-rays, X-rays in 25 fractions), in irradiated groups than in sham-irradiated groups. Among five irradiation regimens, there was heterogeneity in the degree of a difference between irradiated and sham-irradiated groups for two endpoints ($p = 1 \times 10^{-7}$ for crests, $p = 0.046$ for detachment, by chi-square test), but not for one endpoint (large detachment). Among 10 pairs of five irradiation regimens (shown in blue signs), there was a difference in five pairs for two endpoints (crests, detachment) and in four pairs for one endpoint (large detachment). In B6J mice, three endpoints were all higher in aged mice than in young mice. In ApoE$^{-/-}$ mice, two endpoints (crests, large detachment) were higher in aged mice than in young mice. There was no difference in three endpoints between young B6J mice and young ApoE$^{-/-}$ mice. One endpoint (crests) was lower in aged ApoE$^{-/-}$ mice than in aged B6J mice. In ApoE$^{-/-}$ mice, one endpoint (crests) was lower and two endpoints (detachment, large detachment) were higher in mice a fed high-fat diet (HFD) for 16 weeks than in young mice. In ApoE$^{-/-}$ mice, one endpoint (large detachment) was lower in mice fed HFD for 32 weeks than in mice fed HFD for 16 weeks.

A footnote to Figure 3. Among five irradiation regimens, four endpoints (TNF-α, CD68, CD3, and IMT) were higher in all five regimens, two endpoints (CD31 negativity and DAPI negativity) were higher in four regimens (except for chronic γ-rays), two endpoints (eNOS and VE-cadherin) were lower in four regimens (except for chronic γ-rays), one endpoint (TGF-β1) was higher in three regimens (acute X-rays, X-rays in 25 fractions, X-rays in 100 fractions), and one endpoint (F4/80) was higher in three regimens (acute X-rays, X-rays in 25 fractions, chronic X-rays), in irradiated groups than in sham-irradiated groups. Among 10 pairs of five irradiation regimens (shown in blue signs), there was a difference in seven pairs for one endpoint (TGF-β1), six pairs for two endpoints (VE-cadherin and TNF-α), five pairs for two endpoints (CD31 negativity and DAPI negativity), two pairs for three endpoints (eNOS, F4/80 and IMT), and one pair for one endpoint (CD68). In B6J mice as well as in ApoE$^{-/-}$ mice, eight endpoints (CD31 negativity, DAPI negativity, TGF-β1, TNF-α, CD68, F4/80, CD3, and IMT) were higher and two endpoints (eNOS and VE-cadherin) were lower (i.e., there was a difference in all ten endpoints tested) in aged mice than in young mice. Four endpoints (CD31 negativity, DAPI negativity, CD3, and IMT) were higher and two endpoints (eNOS and VE-cadherin) were lower in young ApoE$^{-/-}$ mice than in young B6J mice. Eight endpoints (CD31 negativity, DAPI negativity, TGF-β1, TNF-α, CD68, F4/80, CD3, and IMT) were higher and two endpoints (eNOS and VE-cadherin) were lower (i.e., there was a difference in all ten endpoints tested) in aged ApoE$^{-/-}$ mice than in aged B6J mice. In ApoE$^{-/-}$ mice, five endpoints (CD31 negativity, DAPI negativity, TGF-β1, TNF-α, and CD68) were higher and one endpoint (eNOS) was lower in mice fed a high-fat diet (HFD) for 16 weeks than in young mice. In ApoE$^{-/-}$ mice, three endpoints (TGF-β1, CD3, and IMT) were higher in mice fed HFD for 32 weeks than in mice fed HFD for 16 weeks.

A footnote to Figure 4B–4D. Among four irradiation regimens, one endpoint (intensity of aniline blue stain per unit aortic wall area) was higher in all four regimens, one endpoint (IMT) was higher in three regimens (except for chronic γ-rays), and one endpoint (total intensity of aniline blue stain in the entire aortic wall) was higher in two regimens (acute X-rays, X-rays in 25 fractions), in irradiated groups than in sham-irradiated groups. Among four irradiation regimens, there was heterogeneity in the degree of a difference between irradiated and sham-irradiated groups for three endpoints ($p = 0.044$ for a total intensity of aniline blue stain in the entire aortic wall, $p = 9 \times 10^{-21}$ for crests, $p = 0.006$ for IMT, by chi-square test). Among 6 pairs of four irradiation regimens (shown in blue signs), there was a difference in four pairs for one endpoint (intensity of aniline blue stain per unit aortic wall area), in three pairs for one endpoint (IMT), and in none for one endpoint (total intensity of aniline blue stain in the entire aortic wall). In each of B6J mice and ApoE$^{-/-}$ mice, three endpoints were all higher in aged mice than in young mice. There was no difference in three endpoints between young B6J mice and young ApoE$^{-/-}$ mice. Three endpoints were all higher in aged ApoE$^{-/-}$ mice than in aged B6J mice. In ApoE$^{-/-}$ mice, one endpoint (intensity of aniline blue stain per unit aortic wall area) was higher in mice fed a high-fat diet (HFD) for 16 weeks than in young mice. In ApoE$^{-/-}$ mice, three endpoints were all higher in mice fed an HFD for 32 weeks than in mice fed an HFD for 16 weeks.

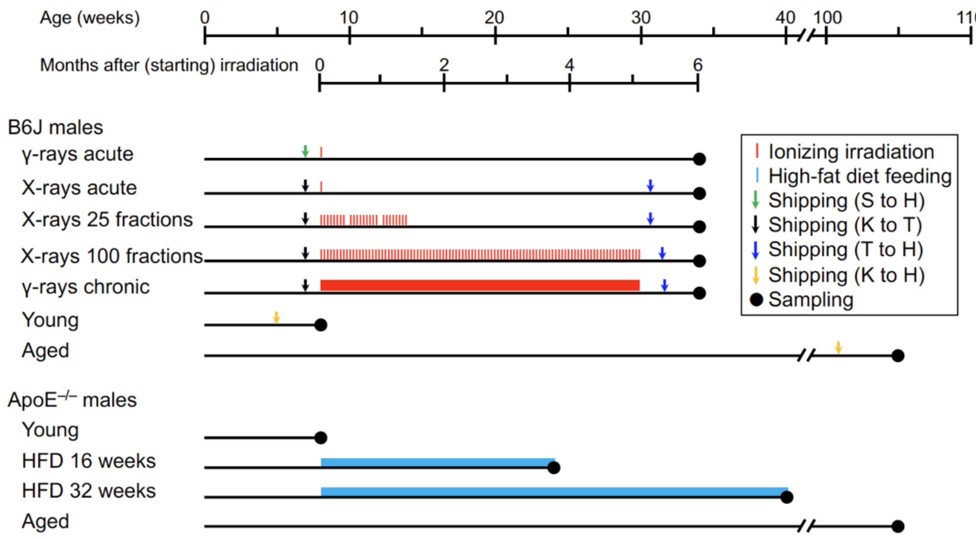

Figure S1. Experimental timelines. This study consists of 12 groups of wild-type C57BL6/J (B6J) male mice and 4 groups of apolipoprotein E-deficient (ApoE$^{-/-}$) mice (details given in Sections 2.1 and 2.2 of the main text). S to H, Shiga to Hiroshima (by car). K to T, Kanagawa to Tokyo (by car). T to H, Tokyo to Hiroshima (by car and air). K to H, Kanagawa to Hiroshima (by car). HFD, high-fat diet. Young, age 8 weeks. Aged, age 104 weeks (2 years).

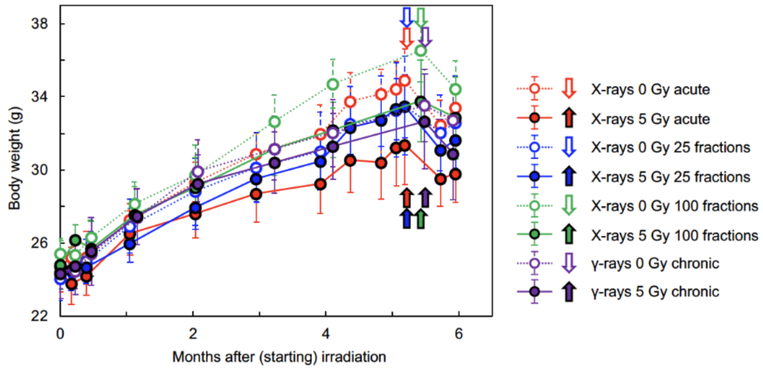

Figure S2. Temporal changes in body weight of irradiated or sham-irradiated B6J mice. Mice were irradiated or sham-irradiated at the age of 8 weeks, and body weight was measured periodically (10 mice/group). There was no difference in body weight among 8 groups at age 8 weeks (i.e., on day 0 when irradiation started) (ANOVA $p = 0.12$). Shipment from Tokyo to Hiroshima (the timing indicated by arrows) led to a decrease in body weight in all 8 groups ($2 \times 10^{-5} < p < 0.02$). Overall body weight was lower in irradiated groups than sham-irradiated groups for four irradiation regimens ($p < 0.04$), but the chi-square test suggests heterogeneity ($p = 1 \times 10^{-10}$) such that the degree of such a decrease was smaller in the two irradiation conditions ($p = 0.04$ for X-rays in 25 fractions, $p = 0.03$ for chronic γ-rays) than in the two irradiation conditions ($p = 3 \times 10^{-29}$ for acute X-rays, $p = 8 \times 10^{-6}$ for X-rays in 100 fractions). Symbols are as indicated on the right.

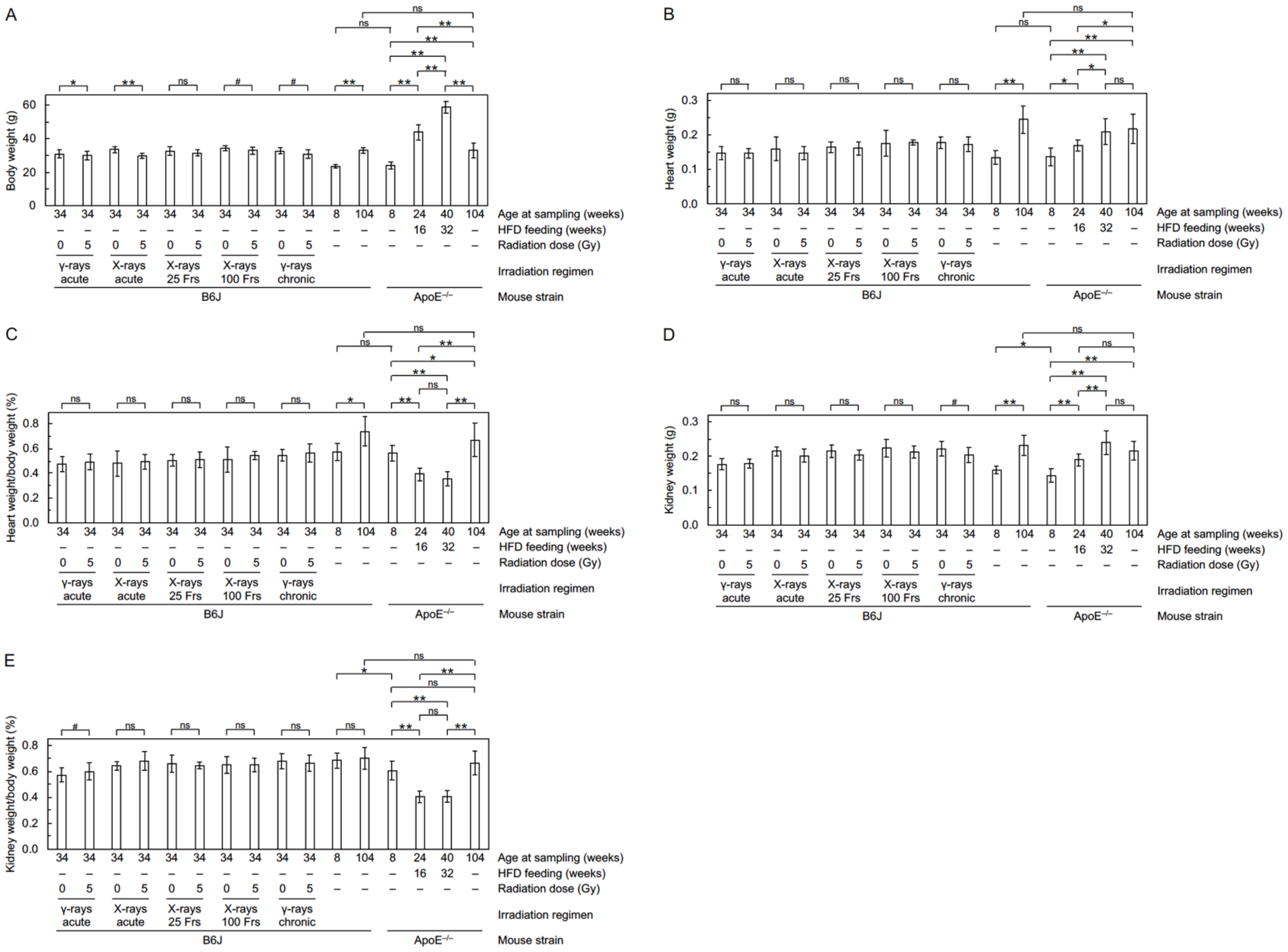

Figure S3. Changes in body weight, heart weight, and kidney weight at sampling. Mice, hearts, and kidneys were weighed at sampling (8–11 mice/group analyzed except for 40 in B6J mice at 6 months after irradiation with 0 Gy or 5 Gy of acute γ-rays and 4 in aged ApoE$^{-/-}$ mice, Welch's *t*-test). (**A**) Body weight. (**B**) Heart weight. (**C**) Heart weight/body weight. (**D**) Kidney weight. (**E**) Kidney weight/body weight. Among five irradiation regimens, there were differences between irradiated and sham-irradiated groups in four regimens (acute γ-rays, acute X-rays, X-rays in 100 fractions, and chronic γ-rays) for body weight, and in one regimen (chronic γ-rays) for kidney weight and for kidney weight/body weight. For any of five endpoints, there was no heterogeneity in the degree of a difference between irradiated and sham-irradiated groups among five irradiation regimens ($0.13 < p < 0.98$ by chi-square test). In both B6J and ApoE$^{-/-}$ mice, four endpoints (body weight, heart weight, heart weight/body weight, kidney weight) were higher in aged mice than in young mice. Two endpoints (kidney weight, kidney weight/body weight) were lower in young ApoE$^{-/-}$ mice than in young B6J mice. There was no difference in five endpoints between aged B6J mice and aged ApoE$^{-/-}$ mice. In ApoE$^{-/-}$ mice, three endpoints (body weight, heart weight, kidney weight) were higher and two endpoints (heart weight/body weight, kidney weight/body weight) were lower in mice fed a high-fat diet (HFD) for 16 weeks than in young mice. In ApoE$^{-/-}$ mice, three endpoints (body weight, heart weight, kidney weight) were higher in mice fed an HFD for 32 weeks than in mice fed an HFD for 16 weeks. Frs, fractions. **, $p < 0.001$. *, $0.001 \le p < 0.05$. #, $0.05 \le p < 0.1$ (marginally significant). ns, $p \ge 0.1$ (nonsignificant).

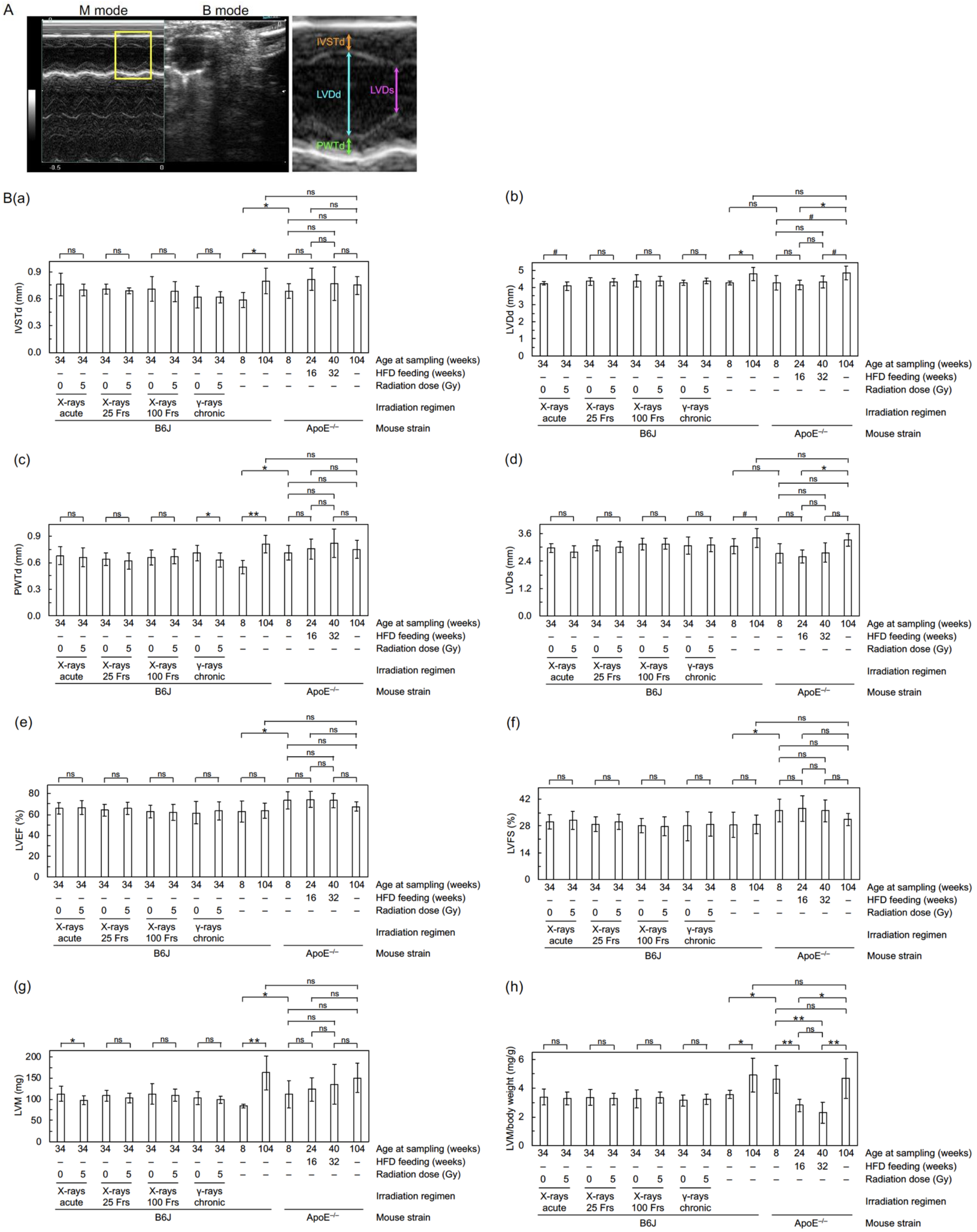

Figure S4. Changes in left ventricular function. (**A**). A representative echocardiographic image (young B6J mouse). A boxed area in an M-mode image is shown at higher magnification on the right. M mode, motion/movement mode. B mode, brightness mode. (**B**). M-mode echocardiographic indices (details given in Section 2.3 of the main text, 8–11 mice/group analyzed except for 4 in aged

ApoE$^{-/-}$ mice, Welch's t-test): (**a**) interventricular septal thickness at end diastole (IVSTd), (**b**) left ventricular dimension at end diastole (LVDd), (**c**) left ventricular posterior wall thickness at end diastole (PWTd), (**d**) left ventricular dimension at end systole (LVDs), (**e**) left ventricular ejection fraction (LVEF), (**f**) left ventricular fractional shortening (LVFS), (**g**) left ventricular mass (LVM), and (**h**) LVM/body weight. Among four irradiation regimens, there were differences between irradiated and sham-irradiated groups only in one regimen for four endpoints (chronic γ-rays for IVSTd and PWTd, acute X-rays for LVDd, and LVM). For any of eight endpoints, there was no heterogeneity in the degree of a difference between irradiated and sham-irradiated groups among four irradiation regimens ($0.26 < p < 0.96$ by chi-square test). In B6J mice, six endpoints (IVSTd, LVDd, PWTd, LVDs, LVM, LVM/body weight) were higher in aged mice than in young mice. In ApoE$^{-/-}$ mice, one endpoint (LVDd) was higher in aged mice than in young mice. Six endpoints (IVSTd, PWTd, LVEF, LVFS, LVM, LVM/body weight) were higher in young ApoE$^{-/-}$ mice than in young B6J mice. There was no difference in eight endpoints between aged B6J mice and aged ApoE$^{-/-}$ mice. In ApoE$^{-/-}$ mice, one endpoint (LVM/body weight) was lower in mice fed a high-fat diet (HFD) for 16 weeks than in young mice, and there was no difference in eight endpoints between mice fed an HFD for 16 weeks and mice fed an HFD for 32 weeks. Frs, fractions. **, $p < 0.001$. *, $0.001 \le p < 0.05$. #, $0.05 \le p < 0.1$ (marginally significant). ns, $p \ge 0.1$ (nonsignificant).

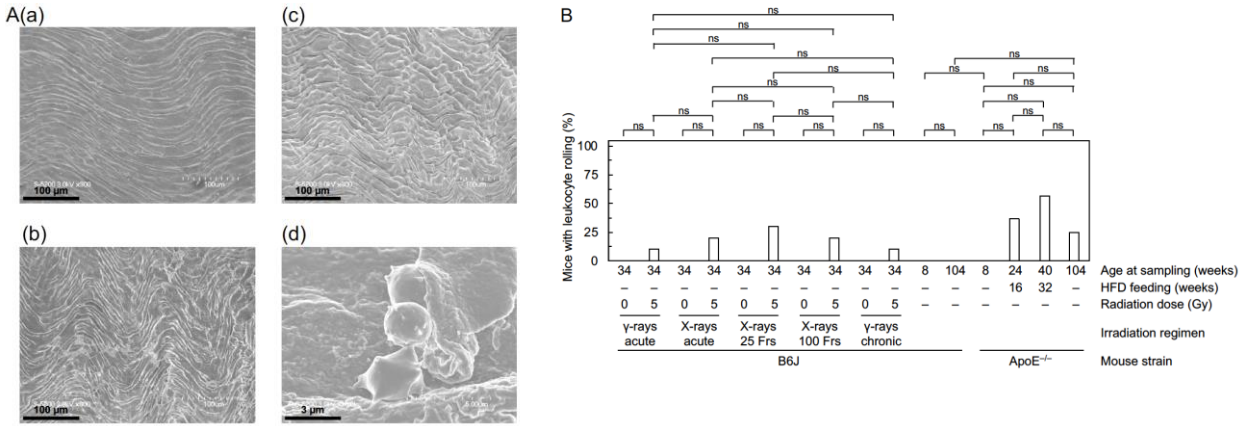

Figure S5. Morphological changes in the aortic endothelium. (**A**). Representative FE-SEM images in B6J mice (all at 6 months after starting irradiation at 5 Gy) of (**a**) flattened endothelium (X-rays in 25 fractions), (**b**) deranged endothelium (acute X-rays), (**c**) cobblestone (or snap pea)-shaped endothelium (X-rays in 100 fractions), and (**d**) endothelium with rolling leukocytes (X-rays in 25 fractions). Scale bars as indicated. (**B**). Quantitative analysis for percentage of mice with leukocyte rolling (7–11 mice/group analyzed except for 4 in aged ApoE$^{-/-}$ mice, Fisher's exact test). There was no inter-group difference in any comparisons. Frs, fractions. The data in Figure S5B for the two B6J groups receiving 0 Gy or 5 Gy of acute γ-rays were taken from the 2020 Cancers paper [1]. ns, $p \ge 0.1$ (nonsignificant).

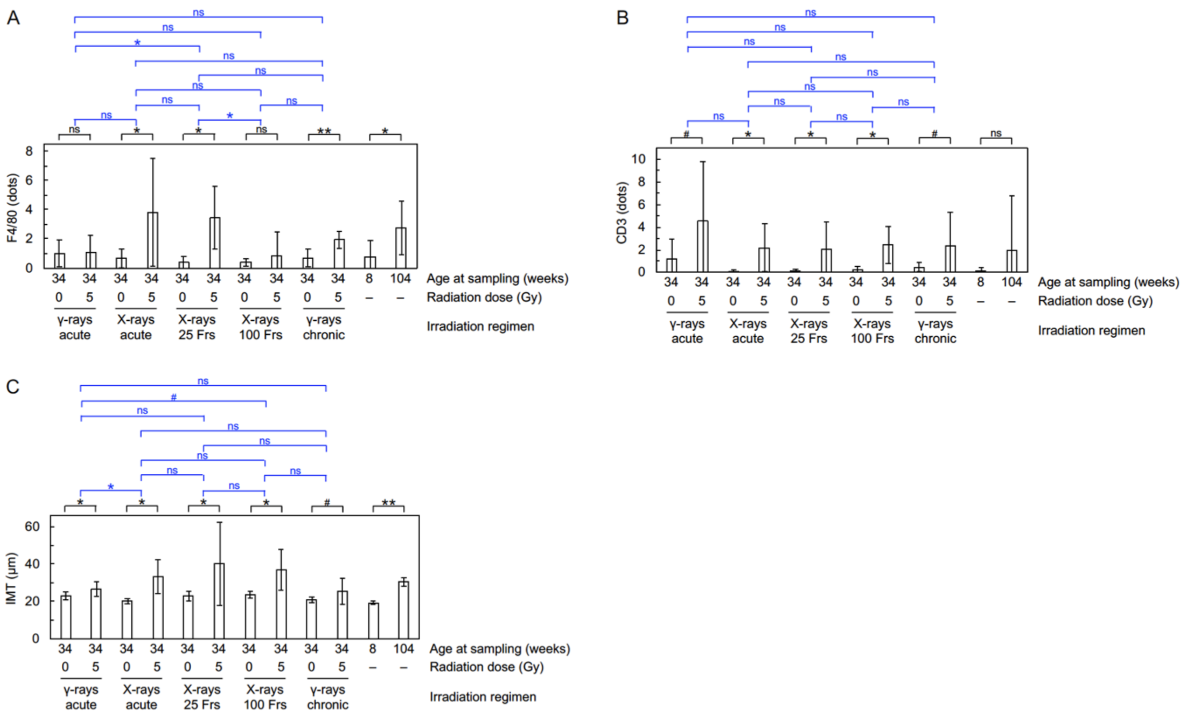

Figure S6. Molecular changes in the aorta of B6J mice. Quantitative analysis for (**A**) F4/80, (**B**) CD3, and (**C**) IMT, which were replotted from the data for B6J mice in Figure 3G, 3H, and 3J, respectively (see legend for details). **, $p < 0.001$. *, $0.001 \le p < 0.05$. #, $0.05 \le p < 0.1$ (marginally significant). ns, $p \ge 0.1$ (nonsignificant).

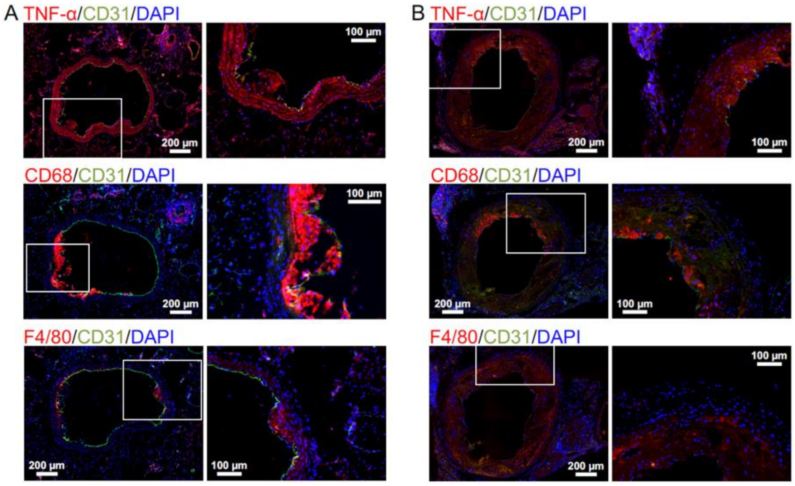

Figure S7. Molecular changes in the aorta of ApoE$^{-/-}$ mice. Representative merged images for double immunofluorescence of CD31 with TNF-α, CD68, or F4/80 in (**A**) ApoE$^{-/-}$ mice fed a high-fat diet for 32 weeks and in (**B**) aged ApoE$^{-/-}$ mice. Scale bars as indicated. Quantitative data are presented in Figure 3. **, $p < 0.001$. *, $0.001 \le p < 0.05$. #, $0.05 \le p < 0.1$ (marginally significant). ns, $p \ge 0.1$ (nonsignificant).

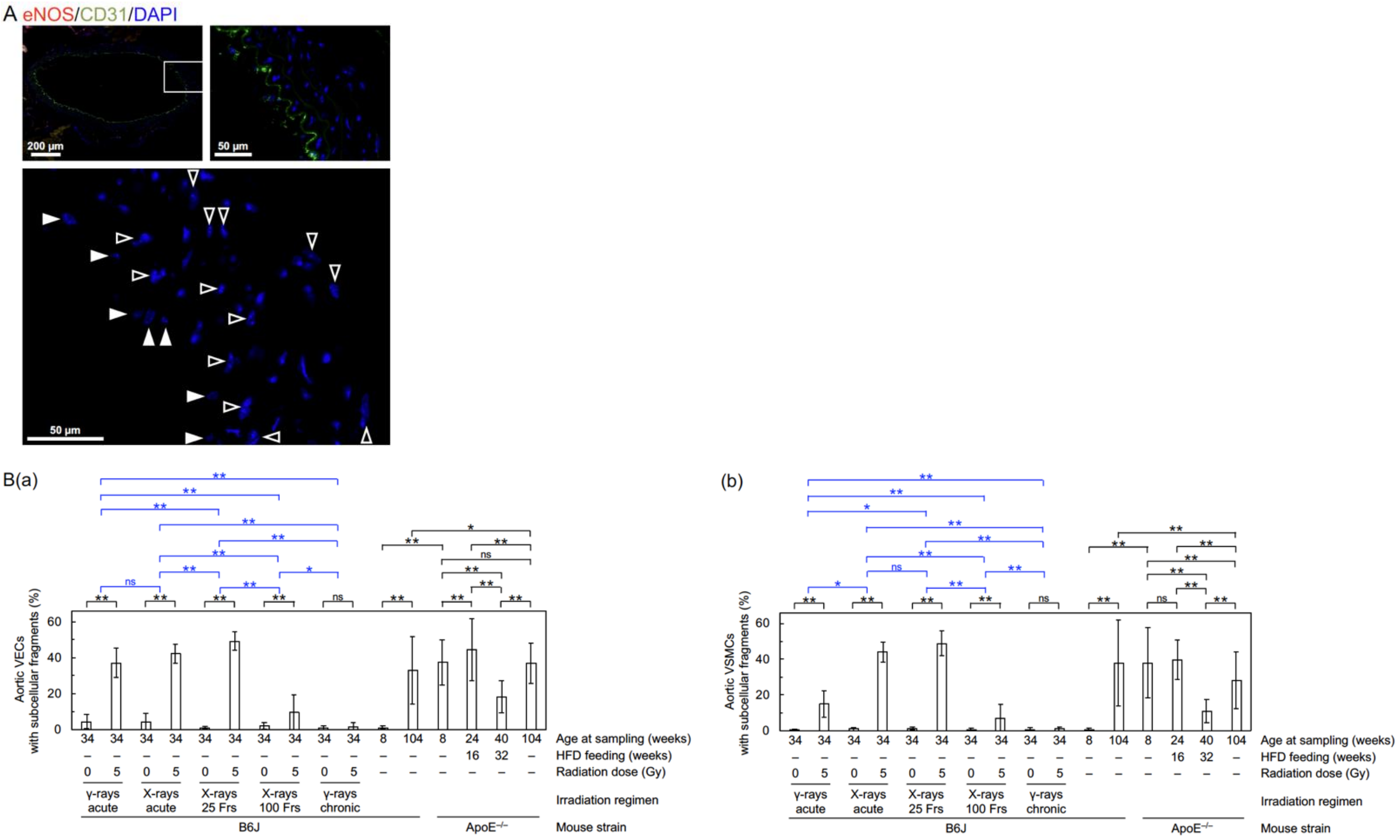

Figure S8. Nuclear changes in the aorta. (**A**) Representative immunofluorescence images for cells with subcellular fragments in the aorta. A boxed area in the upper left panel (a tiled image) is shown at higher magnification in the upper right panel. The upper left panel is the same as the lower left panel in Figure 2A (i.e., B6J mouse at 6 months after starting irradiation with 5 Gy of X-rays in 25 fractions). Likewise, the upper right panel is the same as the lower right panel in Figure 2A, but without eNOS (i.e., the image merged only for CD31 and DAPI). The upper right panel is further enlarged in the lower panel, but without CD31 (i.e., only DAPI). Closed and open arrowheads point to vascular endothelial cells (VECs) with subcellular fragments in the tunica intima and vascular smooth muscle cells (VSMCs) with subcellular fragments in the tunica media, respectively. Scale bars as indicated. (**B**) Quantitative analysis for (**a**) aortic VECs with subcellular fragments and (**b**) aortic VSMCs with subcellular fragments (7–11 mice/group analyzed except for 4 in aged ApoE$^{-/-}$ mice, 120–388 VECs and 306–2230 VSMCs counted/mouse, Wald test). (**a**,**b**) In four out of five irradiation regimens (except for chronic γ-rays), two endpoints (VECs and VSMCs) were both higher in irradiated groups than in sham-irradiated groups. Among 10 pairs of five irradiation regimens (shown in blue signs), there was a difference in eight pairs for one endpoint (VECs) and in nine pairs for the other endpoint (VSMCs). In B6J mice, two endpoints were both higher in aged mice than in young mice. In ApoE$^{-/-}$ mice, one endpoint (VSMCs) was lower in aged mice than in young mice. Two endpoints were both higher in young ApoE$^{-/-}$ mice than in young B6J mice. One endpoint (VECs) was lower and the other endpoint (VSMCs) was higher in aged ApoE$^{-/-}$ mice than in aged B6J mice. In ApoE$^{-/-}$ mice, one endpoint (VECs) was higher in mice a fed high-fat diet (HFD) for 16 weeks than in young mice. In ApoE$^{-/-}$ mice, two endpoints were both lower in mice fed an HFD for 32 weeks than in mice fed an HFD for 16 weeks. Frs, fractions. The data in Figure S8B for the two B6J groups receiving 0 Gy or 5 Gy of acute γ-rays were taken from the 2020 Cancers paper [1]. **, $p < 0.001$. *, $0.001 \le p < 0.05$. #, $0.05 \le p < 0.1$ (marginally significant). ns, $p \ge 0.1$ (nonsignificant).

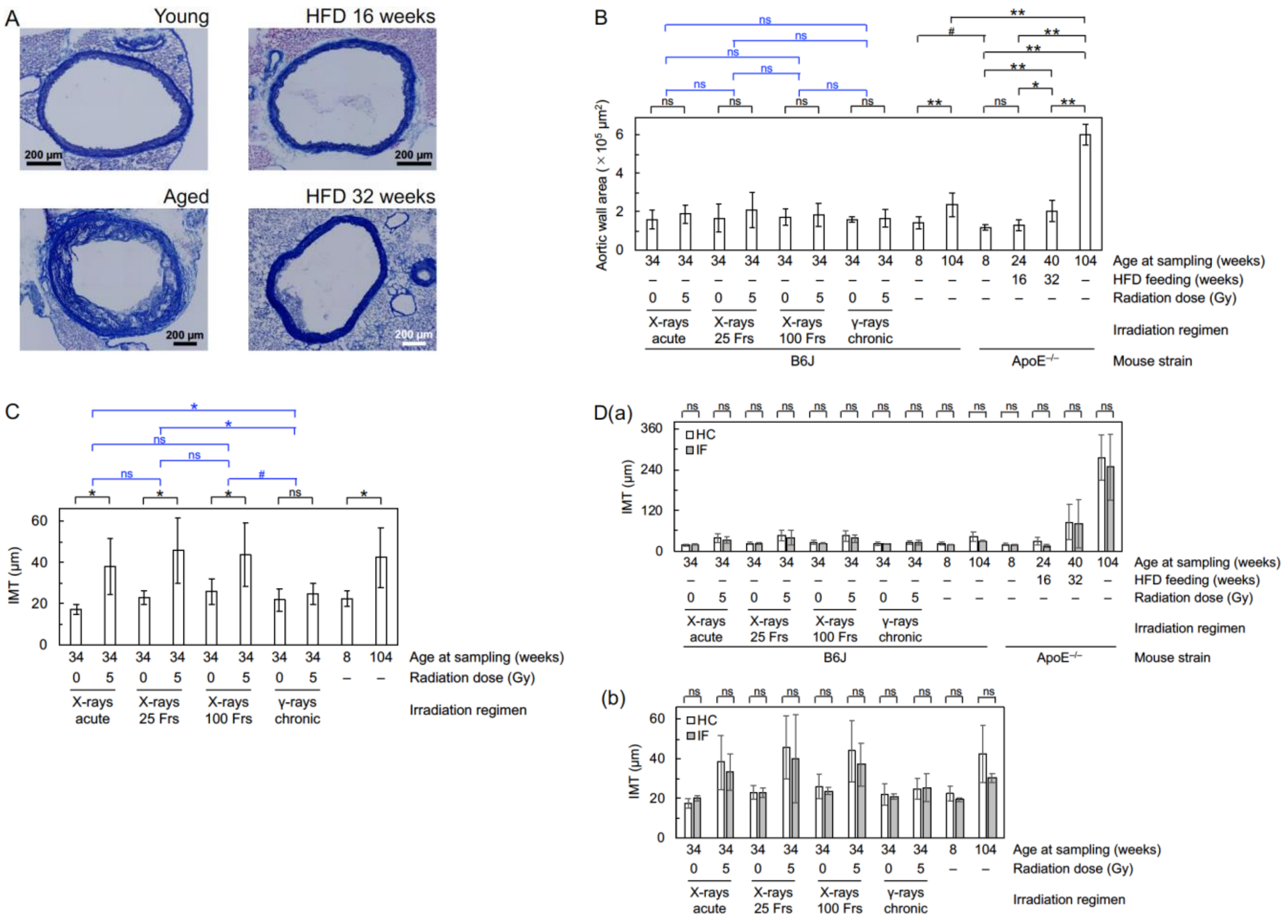

Figure S9. Fibrotic changes in the aorta. (**A**). Representative images for Masson's trichrome staining in ApoE$^{-/-}$ mice (young, aged, high-fat diet feeding for 16 or 32 weeks). Scale bars as indicated. (**B**) Quantitative analysis for aortic wall area (8–10 mice/group analyzed except for 4 in aged ApoE$^{-/-}$ mice, Welch's *t*-test). Among four irradiation regimens, there was no difference between irradiated and sham-irradiated groups, and there was no heterogeneity in the degree of a difference between irradiated and sham-irradiated groups. In each of B6J mice and ApoE$^{-/-}$ mice, the endpoint was higher in aged mice than in young mice. The endpoint was lower in young ApoE$^{-/-}$ mice than in young B6J mice and was higher in aged ApoE$^{-/-}$ mice than in aged B6J mice. In ApoE$^{-/-}$ mice, there was no difference between mice fed a high-fat diet (HFD) for 16 weeks and in young mice, but the endpoint was higher in mice fed an HFD for 32 weeks than in mice fed an HFD for 16 weeks. (**C**). Quantitative analysis for IMT, which was replotted from the data for B6J mice in Figure 4D (see its legend for details). (**D**) Comparison of IMT determined from histochemistry images (open columns) or from immunofluorescence images (filled columns). (**a**) The panel replotted from Figures 3J and 4D (see its legend for details). (**b**) The panel replotted from the data for B6J mice in Figure S9D(a). There was no difference in IMT between in any of the 14 groups (Welch's *t*-test). **, $p < 0.001$. *, $0.001 \le p < 0.05$. #, $0.05 \le p < 0.1$ (marginally significant). ns, $p \ge 0.1$ (nonsignificant).

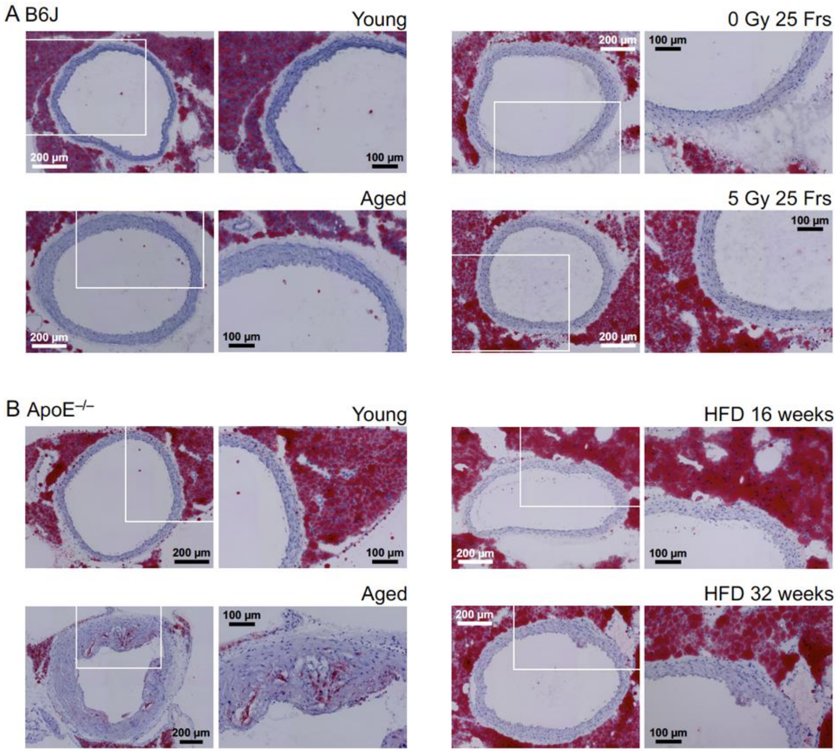

Figure S10. Atherosclerotic plaques in the aorta. Representative images for Oil Red O staining in (**A**) B6J mice (young, aged, at 6 months after staring irradiation with 0 Gy or 5 Gy of X-rays in 25 fractions) and in (**B**) ApoE−/− mice (young, aged, high-fat diet feeding for 16 or 32 weeks). Boxed areas in the left panels (tiled images) are shown at higher magnification in the right panels. Scale bars as indicated.

Supplementary Tables

Table S1. List of 24 endpoints changed in irradiated B6J mice at least in one irradiation regimen (vs sham-irradiated B6J mice).

16 endpoints increased	
	Kidney weight/body weight, mice with detachment, mice with large detachment, CD31 negativity, DAPI negativity, TNF-α, CD68, F4/80, CD3, TGF-β1, IMT-IF, aortic VECs with subcellular fragments, aortic VSMCs with subcellular fragments, total stained intensity in the entire aortic wall area, stained intensity per unit aortic wall area, IMT-HC
8 endpoints decreased	
	Body weight, kidney weight, LVDd, PWTd, LVM, the number of crests/field, eNOS, VE-cadherin

IMT-IF, IMT determined from immunofluorescence images (Figures 3J and S6C). IMT-HC, IMT determined from histochemistry images (Figures 4D and S9C). For details of statistical comparisons in each endpoint, see the respective figure and its legend (Figures 1, 3, 4, S3, S4, S6, S8 and S9).

Table S2. Comparison of multiple endpoints in relation to changes in the aorta by scoring of categorized levels of statistical differences among irradiation regimens.

**Endpoint** [a,b]	**Data presented in**	**Scores for differences among irradiation regimens** [c]									
		**Acute X-rays vs acute γ-rays**	**X-rays 25 Frs vs acute γ-rays**	**Acute γ-rays vs X-rays 100 Frs**	**Acute γ-rays vs chronic γ-rays**	**X-rays 25 Frs vs acute X-rays**	**Acute X-rays vs X-rays 100 Frs**	**Acute X-rays vs chronic γ-rays**	**X-rays 25 Frs vs X-rays 100 Frs**	**X-rays 25 Frs vs chronic γ-rays**	**X-rays 100 Frs vs chronic γ-rays**
The number of crests/field	Figure 1B(a)	0.0	1.0	1.0	1.5	0.0	0.0	0.0	1.5	1.5	0.0
Mice with detachment	Figure 1B(b)	0.0	0.0	0.0	1.5	0.0	1.0	1.0	1.0	1.5	0.0
Mice with large detachment	Figure 1B(c)	0.0	0.0	1.0	1.0	0.0	0.0	0.0	1.5	1.5	0.0
CD31 negativity	Figure 3A	0.0	0.0	0.0	1.5	0.0	0.0	1.5	1.0	1.5	1.5
DAPI negativity	Figure 3B	0.0	0.0	0.0	1.5	0.0	0.0	1.5	1.0	1.5	1.5
eNOS	Figure 3C	0.0	0.0	0.0	1.0	0.0	0.0	1.0	0.0	0.0	0.0
VE-cadherin	Figure 3D	0.0	–1.5	1.0	1.5	0.0	0.0	1.5	0.0	1.5	1.5
TNF-α	Figure 3E	1.5	1.5	0.0	0.0	0.0	1.5	1.5	1.0	1.5	0.0
CD68	Figure 3F	0.0	0.0	0.0	0.0	0.0	0.0	0.0	0.0	1.0	0.0
F4/80	Figures 3G and S6A	0.0	1.0	0.0	0.0	0.0	0.0	0.0	1.0	0.0	0.0
TGF-β1	Figure 3I	1.5	1.5	–1.5	0.0	0.5	0.0	1.0	0.0	1.5	1.0
IMT	Figures 3J and S6C	1.0	0.0	–0.5	0.0	0.0	0.0	0.0	0.0	0.0	0.0
Aortic VECs with subcellular fragments	Figure S8B(a)	0.0	1.5	1.5	1.5	1.5	1.5	1.5	1.5	1.5	1.0
Aortic VSMCs with subcellular fragments	Figure S8B(b)	1.0	1.0	1.5	1.5	0.0	1.5	1.5	1.5	1.5	1.5
Stained intensity per unit aortic wall area	Figure 4C	N.A.	N.A.	N.A.	N.A.	1.5	0.0	1.0	1.5	1.5	0.0

IMT	Figures 4D and S9C	N.A.	N.A.	N.A.	N.A.	0.0	0.0	1.0	0.0	1.0	0.5
Mean score [d]		0.4	0.4	0.3	0.9	0.2	0.3	0.9	0.8	1.2	0.5
Difference judged from each comparison [e]		Acute X-rays > acute γ-rays	X-rays 25 Frs > acute γ-rays	Acute γ-rays > X-rays 100 Frs	Acute γ-rays >> chronic γ-rays	X-rays 25 Frs > acute X-rays	Acute X-rays > X-rays 100 Frs	Acute X-rays >> chronic γ-rays	X-rays 25 Frs >> X-rays 100 Frs	X-rays 25 Frs >> chronic γ-rays	X-rays 100 Frs >> chronic γ-rays
Overall difference judged from all comparisons [f]		X-rays 25 Frs > acute X-rays > acute γ-rays > X-rays 100 Frs >> chronic γ-rays									

Frs, fractions. N.A., data not available. [a] The data for mice with leukocyte rolling (Figure S5B) and CD3 (Figures 3H and S6B) were omitted due to the lack of significant differences in any of 10 pairs among five irradiation regimens. [b] The data for total stained intensity in the entire aortic wall area (Figure 4B) and aortic wall area (Figure S9B) were omitted due to the lack of significant differences in any of 8 pairs among four irradiation regimens. [c] Criteria for "A vs B" comparisons: ** = 1.5 (A > B), * = 1 (A > B), # = 0.5 (A > B), ns = 0 (A = B) ; # = –0.5 (A < B), * = –1 (A < B), ** = –1.5 (A < B). [d] Mean score ranged from 0.22 (X-rays 25 Frs vs acute X-rays) to 1.16 (X-rays 25 Frs vs chronic γ-rays). [e] Criteria for mean scores: > (0.2 < mean score < 0.5), >> (mean score ≥ 0.5). [f] Judged from differences in each of 10 comparisons shown in the second column from the bottom.

Table S3. Comparison of radiation effects in the aorta averaged over 16 endpoints among irradiation regimens.

**Alternative hypotheses [a]**	**One-sided *p* values for equal averaged effects between the given irradiation conditions A vs B [b]**									
	**Acute X-rays vs acute γ-rays**	**X-rays 25 Frs vs acute γ-rays**	**Acute γ-rays vs X-rays 100 Frs**	**Acute γ-rays vs chronic γ-rays**	**X-rays 25 Frs vs acute X-rays**	**Acute X-rays vs X-rays 100 Frs**	**Acute X-rays vs chronic γ-rays**	**X-rays 25 Frs vs X-rays 100 Frs**	**X-rays 25 Frs vs chronic γ-rays**	**X-rays 100 Frs vs chronic γ-rays**
|A| > |B|	5×10^{-3}	3×10^{-4}	6×10^{-3}	3×10^{-4}	8×10^{-2}	4×10^{-6}	1×10^{-10}	3×10^{-7}	9×10^{-12}	7×10^{-6}
|A| < |B|	7×10^{-1}	7×10^{-1}	3×10^{-1}	9×10^{-1}	8×10^{-1}	1×10^{0}	1×10^{0}	1×10^{0}	1×10^{0}	$1 x 10^{0}$
Difference in each comparison [c]	Acute X-rays > acute γ-rays	X-rays 25 Frs > acute γ-rays	Acute γ-rays > X-rays 100 Frs	Acute γ-rays > chronic γ-rays	X-rays 25 Frs > acute X-rays	Acute X-rays >> X-rays 100 Frs	Acute X-rays >> chronic γ-rays	X-rays 25 Frs >> X-rays 100 Frs	X-rays 25 Frs >> chronic γ-rays	X-rays 100 Frs >> chronic γ-rays
Overall difference [d]	X-rays 25 Frs > acute X-rays > acute γ-rays > X-rays 100 Frs >> chronic γ-rays									

Frs, fractions. [a] vs null hypothesis that the absolute values of the radiation effects between the given comparison groups are equal for all endpoints (|A| = |B|). [b] *p* value of one-sided Kolmogorov–Smirnov goodness-of-fit test for the null hypothesis that the distribution of the *p* values from the individual tests (in Table S2) is the standard uniform, which is true with no difference in radiation effects for all endpoints between the given comparison groups. [c] Criteria for *p* values for |A| > |B| : > ($1 \times 10^{-5} \le p < 10^{-1}$), >> ($p < 1 \times 10^{-5}$). [d] Judged from differences in each of 10 comparisons shown in the second column from the bottom.

Table S4. List of 28 endpoints changed in aged B6J mice (vs young B6J mice).

25 endpoints increased	
	Body weight, heart weight, heart weight/body weight, kidney weight, IVSTd, LVDd, PWTd, LVDs, LVM, LVM/body weight, mice with detachment, mice with large detachment, CD31 negativity, DAPI negativity, TNF-α, CD68, F4/80, TGF-β1, IMT-IF, aortic VECs with subcellular fragments, aortic VSMCs with subcellular fragments, total stained intensity in the entire aortic wall area, stained intensity per unit aortic wall area, IMT-HC, aortic wall area
3 endpoints decreased	
	The number of crests/field, eNOS, VE-cadherin

IMT-IF, IMT determined from immunofluorescence images (Figures 3J and S6C). IMT-HC, IMT determined from histochemistry images (Figures 4D and S9C). For details of statistical comparisons in each endpoint, see the respective figure and its legend (Figures 1, 3, 4, S3, S4, S6, S8 and S9).

Table S5. List of endpoints changed in irradiated B6J mice and aged B6J mice.

18 common endpoints
14 endpoints increased commonly
Mice with detachment, mice with large detachment, CD31 negativity, DAPI negativity, TNF-α, CD68, F4/80, TGF-β1, IMT-IF, aortic VECs with subcellular fragments, aortic VSMCs with subcellular fragments, total stained intensity in the entire aortic wall area, stained intensity per unit aortic wall area, IMT-HC
4 endpoints decreased commonly
LVDd, the number of crests/field, eNOS, VE-cadherin
16 differential endpoints
2 endpoints increased only in irradiated B6J mice
Kidney weight/body weight, CD3
4 endpoints decreased only in irradiated B6J mice
Body weight, kidney weight, PWTd, LVM
10 endpoints increased only in aged B6J mice
Body weight, heart weight, heart weight/body weight, kidney weight, IVSTd, PWTd, LVDs, LVM, LVM/body weight, aortic wall area

IMT-IF, IMT determined from immunofluorescence images (Figures 3J and S6C). IMT-HC, IMT determined from histochemistry images (Figures 4D and S9C). For details of statistical comparisons in each endpoint, see the respective figure and its legend (Figures 1, 3, 4, S3, S4, S6, S8 and S9).

Table S6. List of 22 endpoints changed in aged $ApoE^{-/-}$ mice (vs young $ApoE^{-/-}$ mice).

18 endpoints increased
Body weight, heart weight, heart weight/body weight, kidney weight, LVDs, mice with large detachment, CD31 negativity, DAPI negativity, TNF-α, CD68, F4/80, CD3, TGF-β1, IMT-IF, total stained intensity in the entire aortic wall area, stained intensity per unit aortic wall area, IMT-HC, aortic wall area
4 endpoints decreased
The number of crests/field, eNOS, VE-cadherin, aortic VSMCs with subcellular fragments

IMT-IF, IMT determined from immunofluorescence images (Figures 3J and S6C). IMT-HC, IMT determined from histochemistry images (Figures 4D and S9C). For details of statistical comparisons in each endpoint, see the respective figure and its legend (Figures 1, 3, 4, S3, S4, S6, S8 and S9).

Table S7. List of endpoints changed in aged B6J mice and aged ApoE$^{-/-}$ mice.

- 20 common endpoints
  - 17 endpoints increased commonly
    - Body weight, heart weight, heart weight/body weight, kidney weight, LVDs, mice with large detachment, CD31 negativity, DAPI negativity, TNF-α, CD68, F4/80, TGF-β1, IMT-IF, total stained intensity in the entire aortic wall area, stained intensity per unit aortic wall area, IMT-HC, aortic wall area
  - 3 endpoints decreased commonly
    - The number of crests/field, eNOS, VE-cadherin
- 10 differential endpoints
  - 8 endpoints increased only in aged B6J mice
    - IVSTd, LVDd, PWTd, LVM, LVM/body weight, mice with detachment, aortic VECs with subcellular fragments, aortic VSMCs with subcellular fragments
  - 1 endpoint increased only in irradiated ApoE$^{-/-}$ mice
    - CD3
  - 1 endpoint decreased only in irradiated ApoE$^{-/-}$ mice
    - aortic VSMCs with subcellular fragments

IMT-IF, IMT determined from immunofluorescence images (Figures 3J and S6C). IMT-HC, IMT determined from histochemistry images (Figures 4D and S9C). For details of statistical comparisons in each endpoint, see the respective figure and its legend (Figures 1, 3, 4, S3, S4, S6, S8 and S9).

Table S8. List of 17 endpoints changed in young ApoE$^{-/-}$mice (vs young B6J mice).

- 11 endpoints increased
  - IVSTd, PWTd, LVEF, LVM, LVFS, LVM/body weight, CD31 negativity, DAPI negativity, CD3, aortic VECs with subcellular fragments, aortic VSMCs with subcellular fragments
- 6 endpoints decreased
  - Kidney weight, kidney weight/body weight, eNOS, VE-cadherin, IMT-IF, aortic wall area

IMT-IF, IMT determined from immunofluorescence images (Figures 3J and S6C). For details of statistical comparisons in each endpoint, see the respective figure and its legend (Figures 1, 3, 4, S3, S4, S6, S8 and S9).

Table S9. List of 17 endpoints changed in aged ApoE$^{-/-}$ mice (vs aged B6J mice).

- 13 endpoints increased
  - IVSTd, PWTd, LVEF, LVM, LVFS, LVM/body weight, CD31 negativity, DAPI negativity, TGF-β1, CD3, aortic VECs with subcellular fragments, aortic VSMCs with subcellular fragments, total stained intensity in the entire aortic wall area, stained intensity per unit aortic wall area, IMT-HC, aortic wall area
- 4 endpoints decreased
  - The number of crests/field, eNOS, VE-cadherin, aortic VSMCs with subcellular fragments

IMT-HC, IMT determined from histochemistry images (Figures 4D and S9C). For details of statistical comparisons in each endpoint, see the respective figure and its legend (Figures 1, 3, 4, S3, S4, S6, S8 and S9).

Table S10. List of 17 endpoints changed in ApoE$^{-/-}$ mice fed HFD for 16 weeks (vs young ApoE$^{-/-}$ mice).

12 endpoints increased
Body weight, heart weight, heart weight/body weight, mice with detachment, mice with large detachment, CD31 negativity, DAPI negativity, TGF-β1, TNF-α, CD68, aortic VECs with subcellular fragments, total stained intensity in the entire aortic wall area
5 endpoints decreased
Kidney weight, kidney weight/body weight, the number of crests/field, eNOS, LVM/body weight

For details of statistical comparisons in each endpoint, see the respective figure and its legend (Figures 1, 3, 4, S3, S4, S6, S8 and S9).

Table S11. List of 13 endpoints changed in ApoE$^{-/-}$ mice fed HFD for 32 weeks (vs ApoE$^{-/-}$ mice fed HFD for 16 weeks).

10 endpoints increased
Body weight, heart weight, heart weight/body weight, CD3, TGF-β1, IMT-IF, total stained intensity in the entire aortic wall area, stained intensity per unit aortic wall area, IMT-HC, aortic wall area
3 endpoints decreased
Mice with large detachment, aortic VECs with subcellular fragments, aortic VSMCs with subcellular fragments

IMT-IF, IMT determined from immunofluorescence images (Figures 3J and S6C). IMT-HC, IMT determined from histochemistry images (Figures 4D and S9C). For details of statistical comparisons in each endpoint, see the respective figure and its legend (Figures 1, 3, 4, S3, S4, S6, S8 and S9).

Table S12. List of 14 endpoints changed in aged ApoE$^{-/-}$ mice (vs ApoE$^{-/-}$ mice fed HFD for 32 weeks).

12 endpoints increased
Heart weight/body weight, kidney weight/body weight, LVDd, LVM/body weight, CD68, F4/80, IMT-IF, aortic VECs with subcellular fragments, aortic VSMCs with subcellular fragments, total stained intensity in the entire aortic wall area, IMT-HC, aortic wall area
2 endpoints decreased
Body weight, the number of crests/field

IMT-IF, IMT determined from immunofluorescence images (Figures 3J and S6C). IMT-HC, IMT determined from histochemistry images (Figures 4D and S9C). For details of statistical comparisons in each endpoint, see the respective figure and its legend (Figures 1, 3, 4, S3, S4, S6, S8 and S9).

References

1. Hamada, N.; Kawano, K.I.; Yusoff, F.M.; Furukawa, K.; Nakashima, A.; Maeda, M.; Yasuda, H.; Maruhashi, T.; Higashi, Y. Ionizing Irradiation Induces Vascular Damage in the Aorta of Wild-Type Mice. *Cancers* **2020**, *12*, 3030. https://doi.org/10.3390/cancers12103030.
